# Impact of shipping emissions regulation on urban aerosol composition changes revealed by receptor and numerical modelling

**DOI:** 10.1038/s41612-023-00364-9

**Published:** 2023-05-29

**Authors:** Eunhwa Jang, Seongwoo Choi, Eunchul Yoo, Sangmin Hyun, Joongeon An

**Affiliations:** 1 Busan Metropolitan City Institute of Health and Environment, 120, Hambakbong-ro, 140beon-gil, Buk-gu, Busan, 46616 Republic of Korea; 2 Marine Environmental Research Center, Korea Institute of Ocean Science and Technology, 385, Haeyang-ro, Yeongdo-gu, Busan, 49111 Republic of Korea; 3 Risk Assessment Research Center, Korea Institute of Ocean Science and Technology, Geoje, 53201 Republic of Korea

**Keywords:** Environmental impact, Environmental monitoring

## Abstract

Various shipping emissions controls have recently been implemented at both local and national scales. However, it is difficult to track the effect of these on PM_2.5_ levels, owing to the non-linear relationship that exists between changes in precursor emissions and PM components. Positive Matrix Factorisation (PMF) identifies that a switch to cleaner fuels since January 2020 results in considerable reductions in shipping-source-related PM_2.5_, especially sulphate aerosols and metals (V and Ni), not only at a port site but also at an urban background site. CMAQ sensitivity analysis reveals that the reduction of secondary inorganic aerosols (SIA) further extends to inland areas downwind from ports. In addition, mitigation of secondary organic aerosols (SOA) in coastal urban areas can be anticipated either from the results of receptor modelling or from CMAQ simulations. The results in this study show the possibility of obtaining human health benefits in coastal cities through shipping emission controls.

## Introduction

Concerns over the contribution of air pollutants from the shipping sector to global emissions have been widely reviewed^1–4^. Emissions from oceangoing ships are estimated to be 5–8% and 15% of global anthropogenic sulphur oxides (SOx) and nitrogen oxides (NOx), respectively^1^. On average, 70% of air pollutants from shipping activities are emitted within 400 km of coastlines, indicating the potential significance of the air pollution burden from ships on coastal cities and global background particulate matter levels^5^. It was estimated that the vessel emissions associated with manoeuvring and hoteling in the harbour of Los Angeles contributed to 1.4% of the primary PM_2.5_ observed 80 km inland^6^. The contributions from shipping emissions have been estimated at 1**–**14% of PM_2.5_ concentrations observed in European coastal regions, and they account for up to 25% for primary PM_2.5_ in hotspot areas^1,2,5^. The shorter lifetime of NOx inside the ship emissions plume (~1.8 hours) compared to typical ambient marine conditions (~6.6 h at noontime) may result in enhanced levels of nitrate aerosols (e.g., NH_4_NO_3_ and NaNO_3_) near shipping lanes^7^. As well, the accelerated conversion rate of sulphur dioxide (SO_2_) into sulphate (SO_4_^2−^) in shipping plumes with metallic elements (emitted from the combustion of heavy fuel oils) that act as catalysts in oxidation processes, may explain elevated levels of sulphate aerosols (e.g., (NH_4_)_2_SO_4_ and NH_4_HSO_4_) in the marine boundary layer^8,9^. Additionally, transported air masses enriched in SO_2_ with favourable atmospheric conditions (high relative humidity) for SIA formation may result in elevated sulphate aerosols near coastal cities^10,11^.

The significant impact of shipping emissions not only on air quality but also on adverse health outcomes such as cardiovascular disease, respiratory disease and premature death have been well documented^4,12–17^. According to an expert survey from the WHO-HRAPIE project, shipping emissions have been considered an important source category in regard to health at the global scale, owing to the growth of air pollution associated with increasing international trade and the use of low-quality fuels that result in aerosols containing toxic elements^16,18–20^. The concerns over enhanced metal levels in aerosols, especially vanadium (V) and nickel (Ni) in harbour regions have been outlined^21^, as shipping plumes with relatively high acidity can increase the bioavailability of these metallic species^8,22–24^. The high potential of health risks from toxic organic chemicals associated with vessel exhaust emissions, such as polycyclic aromatic hydrocarbons (PAHs) and polychlorinated dibenzo-p-dioxins and polychlorinated dibenzofurans (PCDD/Fs), have also been addressed^5,25^, and elevated nano-particles, originating from shipping exhaust emissions in coastal regions have been raised as concerns^4,19,26,27^.

Due to the reported adverse health effects of air pollution from ships, there has been a sustained effort to mitigate shipping emissions at the global level under the International Convention for the Prevention of Pollution from Ships (MARPOL). Improving fuel consumption efficiencies through adopting new technologies in new ship designs is one of the recent efforts. As well, switching to cleaner fuels from heavy fuel oil (HFO) or installing scrubbers are other options for reducing air pollutants. Concurrently, many port authorities have prepared voluntary incentive schemes to help operators to reduce shipping emissions near ports^5^. The International Maritime Organisation (IMO) recently implemented regulations on shipping emissions, where the sulphur (S) content of ship’s fuel oil is required to be reduced to 0.5% from 3.5% as of January 2020. Additionally, the stricter regulations have been extended to all ships in designated Emission Control Areas (ECA) either by controlling shipping fuel oils with less than the 0.1% S limit or by adopting voluntary speed reduction policies.

An exact quantitative understanding of source-specific contributions to observed pollution levels is paramount for tracking the effectiveness of implementing regulations on targeted sources, and for designing efficiency control strategies for pollution. With regard to the toxicity issue associated with PM_2.5_ from the maritime sector, recent studies have adopted various source apportionment tools (i.e., either receptor models or numerical models with particulate matter source apportionment technology tools) to identify and quantify shipping-source-related PM_2.5_ in urban coastal areas^27–30^. However, most receptor modelling studies in harbour regions have focused on chemically resolved PM_2.5_ data. Currently, there is only limited in-depth source apportionment analysis of gas-phase PM precursors with high temporal resolution at port regions, where a detailed understanding of secondary inorganic/organic components in PM_2.5_ attributable to shipping emissions may be achieved.

In this work, we analyse the impact of shipping emission regulations on air quality with a particular focus on the chemical composition of PM_2.5_ in Busan, the world’s top 10 container port cities (http://www.marinetraffic.com)^31,32^, where the shipping sector accounts for 45% of primary PM_2.5_ emissions in 2019^33^. Application of PMF to three extensive datasets (PM_2.5_ urban background dataset of 2019–2021, PM_2.5_ port dataset of 2019–2021 and Selected Ion Flow Tube Mass Spectrometry (SIFT-MS) port dataset of 2020–2021) allows quantitative identification of the impact of implementing limits for shipping emissions on the aerosol composition. In addition, scenario analysis using SMOKE-WRF/FNL-CMAQ simulations identifies the changes in the spatial distribution of major chemical component in PM_2.5_, arising from regulations on shipping emissions. The results of this study show that the health burden of air pollution can be greatly reduced in coastal cities through shipping emissions management.

## Results

### Trends of PM precursors and major PM_2.5_ chemical components

**Fig. 1:**
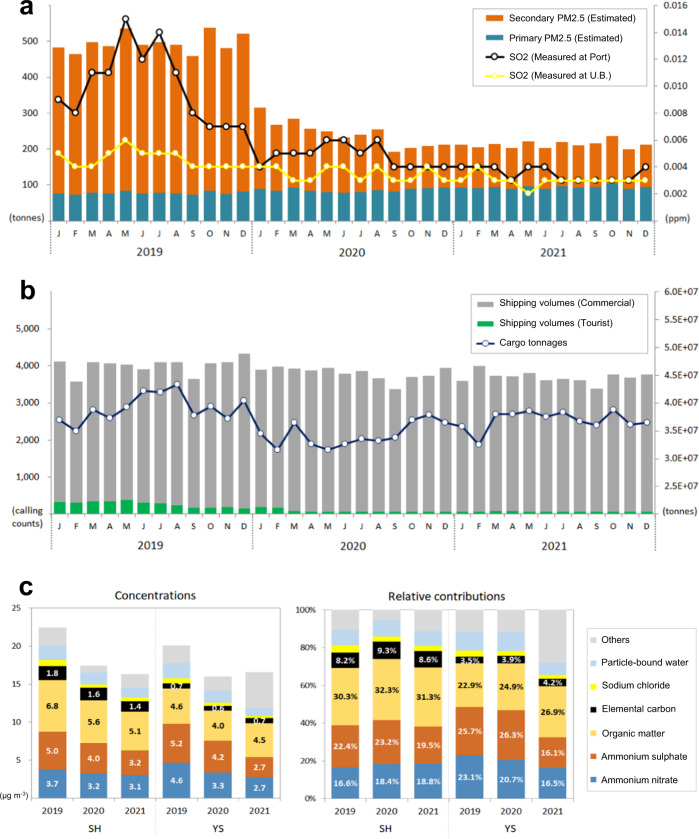
Shipping emissions estimates of PM_2.5_ and PM precursor observations from 2019 to 2021. **a** Bar charts show PM_2.5_ emission estimates from shipping sector in Busan, derived by calculating fuel consumptions based on AIS information; and line charts indicate monthly mean SO_2_ observations at 2 port AQM sites and 28 urban background AQM sites. **b** Bar charts indicate the number of ships entering the port; and line chart shows the total cargo tonnages. **c** Annual variations of reconstructed PM_2.5_ mass concentrations derived from the PM_2.5_ speciation network and their relative contributions to PM_2.5_ level at Sin-Hang and at Yeon-San.

Phasing in the introduction of stricter standards for the S content of marine fuel is described in Supplementary Fig. 1. As shown in Fig. 1a and Supplementary Fig. 2, the monthly mean SO_2_ concentrations at two air quality monitoring (AQM) sites adjacent to the port (hereafter referred to as the port site) in Busan, had been sharply decreasing for several months before the global regulations on S content in shipping fuel oil started in January 2020 (from 3.5 to 0.5%). A comparable declining trend in monthly SO_2_ levels was also observed at urban background sites where hourly measurements at 28 AQM sites in inland Busan were used but to a lesser extent than port sites (SO_2_ reduction rates for 6 months before implementing low-S fuel policy were 71.4% and 20.0% for port sites and urban background sites, respectively).

Figure 1b shows the number of ships calling at all ports in Busan from 2019 to 2021 and the total tonnage of cargo (import + export). The tourist industry seems to have been greatly affected by a 74.4% drop in the number of passenger ships calling (845 in 2021 from 3296 in 2019) since the COVID-19 outbreak; but this was not the case in the commercial shipping sector. The number of cargo vessel calls decreased by only 3.1% (44,828 in 2019 to 43,452 in 2021), and the shipping trade in terms of cargo tonnage decreased by 5.6% (from 469 million tonnes in 2019 to 443 million tonnes in 2020). Based on the fact that the COVID-19 pandemic has minimal impact on the volume of international trade in Korea^31^ and that cargo vessels contribute overwhelmingly to SO_2_ emissions in the Busan shipping sector^33^, changes in SO_2_ emissions from shipping sector in Busan are expected to be minimal during the study period. However, in fact, the concentrations of SO_2_ at port sites in 2021 decreased significantly compared to 2019. Therefore, it can be concluded that the change in shipping traffics was not a major factor in the decrease in SO_2_ emissions over Busan from 2019 to 2021.

The steep reduction of ambient SO_2_ concentrations in Busan after August 2019 seems to be the result of either the predominant wind direction during monsoon climate, in which westerly winds in the cold season leads to reduced advection of air pollutants from the ocean to interior regions; or by the switch to cleaner fuels or pre-installation of scrubbers on international cargo vessels in advance of the regulation date. The stricter requirement of S content in shipping fuels from September 2020 (from 0.5 to 0.1%) for both international and domestic vessels at berth in Busan ports appears to be a reasonable explanation for the concurrent decrease of SO_2_ concentrations at two port sites during the same period. Additional S regulation applied to manoeuvring domestic vessels from January 2021 (3.5–0.5%) might contribute further to the reduction in atmospheric SO_2_ levels in 2021. However, the fact that international cargo ships are the dominant vessel type at Busan ports may be the reason for relatively less significant reductions in both PM_2.5_ emission estimates and observed SO_2_ levels in 2021, compared to values in 2020.

In brief, over the 3 years from 2019 to 2021, the annual mean SO_2_ concentrations in the summer (JJA), which are subject to a relatively large impact from shipping emissions, were 12.3 ppb, 5.7 ppb and 3.3 ppb at port sites and 5.0 ppb, 3.7 ppb and 3.0 ppb at urban background sites in Busan. This considerable reduction in PM precursors (i.e., SO_2_) might be associated with recent international regulations on S content in shipping fuels. Therefore, changes in the chemical composition of aerosols in Busan could be anticipated in the post-S-reduction period.

In Fig. 1c, it can be seen that the concentrations of most major chemical components of PM_2.5_, estimated using the mass reconstruction method^34^, were diminished from 2019 to 2021. The reduction in ammonium sulphates (e.g., (NH_4_)_2_SO_4_, (NH_4_)_3_H(SO_4_)_2_, NH_4_HSO_4_) was most evident at both the port site (Sin-Hang) and at the urban background site (Yeon-San). Detailed variations in individual species over 3 years are additionally described in Supplementary Fig. 2. When considering the relative contribution (%) of major components to PM_2.5_ mass, distinctive changes in the chemical composition of aerosols was observed year-to-year in Busan, especially at the urban background site. The contributions of ammonium sulphates in PM_2.5_ were clearly decreased from 25.7% in 2019 to 16.1% in 2021 at Yeon-San, while they are 22.4% in 2019 and 19.5% in 2021 at Sin-Hang. This may be explained by the significantly reduced PM precursor emissions (i.e., SO_2_) through regulations on fuel oils for ships, leading to reductions in secondary sulphate aerosols formed during transport from near ports to inland urban areas. Meanwhile, a slightly enhanced contribution of ammonium sulphate to PM_2.5_ seen in 2020 was associated with 5-day acute air pollution events in August arising from the long-range transport of air masses containing volcanic ash that had erupted in the Kobe area in Japan. It was reported that elevated volcanic SO_2_ emissions could result in the formation of significant quantities of sulphate aerosols during travel^35,36^.

We may infer that the sudden reduction of sulphate aerosols in Busan since 2020 is associated with the decrease in PM precursor emissions through the regulations on shipping fuel oils starting January 2020. However, advanced source apportionment analysis is necessary to segregate the source-specific contributions to PM_2.5_ constituents, which allows an in-depth quantitative assessment of the impact of implementing the marine pollution control policy, on air quality. In the following sections, the PMF technique was adopted to identify and quantify the impact of shipping emission regulations on aerosol chemical composition.

### Source apportionment of PM_2.5_

**Fig. 2:**
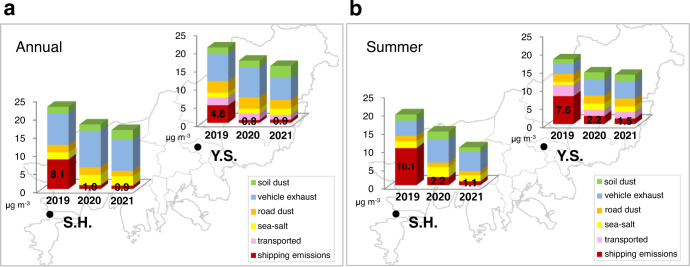
PM_2.5_ mass concentrations at Sin-Hang and at Yeon-San, as a function of PMF analysis. **a** Annual mean concentrations. **b** Mean concentration during the summer.

PMF source apportionment results at Sin-Hang and at Yeon-San and the source-specific contribution to annual PM_2.5_ levels are shown in Supplementary Fig. 3 and Fig. 2, respectively. In 2019, shipping-related sources were significantly responsible for PM_2.5_ mass, not only at Sin-Hang (8.1 μg m^−3^, 36.0%) but also at inland Yeon-San (4.8 μg m^−3^, 23.2%) (see Fig. 2a). Further, this contribution was considerably increased in summer owing to the monsoon climate (Sin-Hang: 10.1 μg m^−3^, 52.3%; Yeon-San: 7.6 μg m^−3^, 43.0%) (see Fig. 2b). These results are quite comparable to the most recently published national emission estimates in which the shipping-source sector is responsible for 45% of annual PM_2.5_ emissions in Busan (2523 tonnes in 2019)^33^. Meanwhile, from 2020 onward, the shipping-source contribution to PM_2.5_ had been considerably reduced. Annual PM_2.5_ mass associated with shipping sources was reduced by 88.9% (from 8.1 μg m^−3^ in 2019 to 0.9 μg m^−3^ in 2021) and 81.3% (from 4.8 μg m^−3^ in 2019 to 0.9 μg m^−3^ in 2021) at Sin-Hang and Yeon-San, respectively. It is likely that these large reductions in shipping-related PM_2.5_ mass in the ambient air are associated with stepwise regulations on shipping emissions from January 2020, although information on national emission estimates for the period of post-S reduction, i.e., from 2020 are not available. Sometimes, the old emission factors may not provide an appropriate explanation for recent atmospheric environments. However, PMF source apportionment results as described in this study could provide current information on sources requiring further control strategies to achieve reductions in PM_2.5_ and also for tracking the effect of recent policies on air quality. A timely and quantitative assessment of the impact of air quality policies based on these observations might be extremely useful to policymakers for setting priorities.

**Fig. 3:**
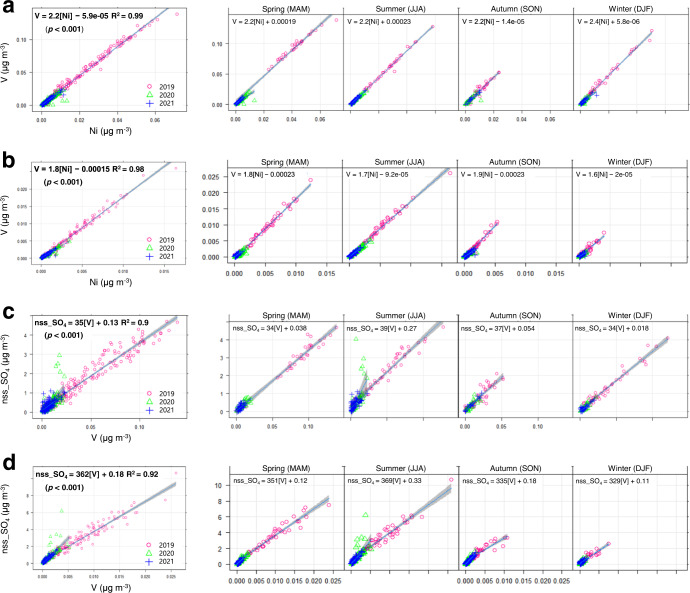
Relationships between shipping emission tracers derived from PMF shipping-source profile. **a** Correlation between V and Ni at Sin-Hang and **b** at Yeon-San. **c** Correlation between nss-SO_4_^2−^ and V at Sin-Hang and **d** at Yeon-San.

In Fig. 3, concentrations segregated as a function of PMF analysis between 2019 and 2021 were assessed with a particular focus on trace elements within shipping exhaust emissions (i.e., V, Ni and non-sea salt sulphate (nss-SO_4_^2−^), where [nss-SO_4_^2−^] is estimated as [SO_4_^2−^] − 0.25 × [Na^+^])^1,37^. At Sin-Hang, the shipping-source-related concentrations ranged from (0.0–138.4) × 10^−3 ^μg m^−3^, (0.0–71.5) × 10^-3 ^μg m^−3^, (0.0–4.7) μg m^−3^ for V, Ni and nss-SO_4_^2−^, respectively. And they ranged (0.0–26.0) × 10^−3 ^μg m^−3^ for V, (0.0–16.4) × 10^−3 ^μg m^−3^ for Ni and (0.0–10.9) μg m^−3^ for nss-SO_4_^2−^ at Yeon-San. As expected, relatively higher levels of metals (i.e., V and Ni) contained in PM_2.5_ were observed adjacent to ports (Fig. 3a), compared to values at the inland site (Fig. 3b). On the other hand, interestingly, much higher concentrations of airborne nss-SO_4_^2−^ were seen inland (Fig. 3d) than at the port (Fig. 3c). This seems to be associated with secondary formation of sulphate aerosols, where freshly emitted gas-phase PM precursor (i.e., SO_2_) is oxidised to particulate SO_4_^2−^ during transport from the port region to inland areas.

With regards to annual trends for shipping trace elements, apparently reduced levels were observed for all three species following S-reduction (2020–2021) both at port site and urban background site in Busan. Zhang et al.^38^ reported that V is linearly reduced depending on S content in shipping fuel oils, while a linear relationship is expected to be unclear for Ni^39^, as Ni in urban air is determined by diverse sources, such as crustal and industrial emissions. Xiao et al.^9^ concluded that V is not an appropriate shipping emission indicator for the period of switching to lower-S fuels. However, in our study, an optimised solution in PMF analysis could be obtained when applied to a PM_2.5_ speciation dataset covering both periods (pre-regulation of 2019 and post-regulation of 2020–2021). Our PMF-resolved results reflected that moving to a cleaner fuel in the shipping sector likely contributes significantly to an improvement of air quality in coastal cities, particularly in terms of inorganic aerosols and metallic components, such as V and Ni, in PM_2.5_. When considering seasonal variations of these compounds, clearly enhanced levels were observed in the warm season. A comparable seasonality for shipping trace metals in coastal cities has been reported^39^. According to CMAQ modelling simulations by Zhao et al., the highest enhancement of V and Ni in surface air attributable to shipping emissions in the spring, was estimated in the Korean Peninsula.

The correlation coefficients among concentrations of three markers obtained by PMF shipping-source profile, were relatively high (*r*^2^ > 0.9), compared to the plots for raw measurements (see Supplementary Fig. 4). When the spatial distribution of ratios between shipping tracers was assessed, a slightly enhanced ratio of V to Ni (V/Ni) in PM_2.5_ was observed at Sin-Hang (2.2, see Fig. 3a), compared to Yeon-San (1.8, see Fig. 3b). The V/Ni ratios obtained in this study were comparable to those reported by others. Manousaks et al. reported that the V/Ni ratio in PM_2.5_, derived from the PMF shipping-source is 3.0^40^. It was shown that the V/Ni ratios were 2.3–3.5 and 2.06 for shipping exhausts and crustal sources, respectively^1,41^. The V/Ni ratio measured in shipping fuel oil was about 2.5^42^. Meanwhile, interestingly the ratios of nss-SO_4_^2−^ to V (nss-SO_4_^2−^/V) in PM_2.5_ were apparently distinguishable between the two sites, with mean ratios of 35 and 362 at Sin-Hang and Yeon-San, respectively (see Fig. 3c, d). The fact that ammonium sulphates are major secondary inorganic components in PM_2.5_ and formed during transport may result in the developed levels of SO_4_^2−^ in inland areas. Therefore, it may be inferred that around 90% of nss-SO_4_^2−^ in PM_2.5_ is attributable to secondary formation at Yeon-San, approximately 6 km to the north of the port regions, while only 10% of nss-SO_4_^2−^ is associated with freshly emitted emissions, under the following assumptions: chemical reaction of V contained in PM_2.5_ is minor during transport from near port regions to inland location; and the use of PMF-resolved ratio between two components (i.e., nss-SO_4_^2−^/V) can normalise the shipping plume dilution effect. It was demonstrated that the nss-SO_4_^2−^/V ratio measured from shipping exhausts ranged from 11 to 27^41^, and the higher ratio of 200–400 was obtained from inland urban aerosols in the summer, associated with shipping as the source^1^. The significant contribution from shipping emissions to secondary PM_2.5_ formation has been outlined in other studies. At Yangshan Harbour in China, the shipping-source contribution to annual PM_2.5_ was estimated as 1.02 μg m^−3^ using a PMF-based method, and this value was higher by one order of magnitude than that of primary PM_2.5_ estimated from V-based method (0.10 μg m^−3^)^16,43^. These results have significant implications in terms of the wisdom of controlling shipping emissions in coastal cities to mitigate PM_2.5_ levels, which can ultimately provide human health benefits.

With regards to PM_2.5_ carbonaceous fractions (i.e., OC and EC) derived from PMF shipping emission factors, considerably reduced concentrations were observed during the period of post-S-reductions (see Supplementary Fig. 5). The emissions of not only S, but also other pollutants seem to be concurrently reduced through combustion of cleaner fuel. In addition, the reductions in the consumption of lubricating oils required to neutralise the acidic products thereby preventing engine corrosion could contribute to mitigating emissions of the carbonaceous fractions^44,45^. Interestingly, this apparent annual reduction of carbonaceous fractions was not seen in PMF traffic emissions.

### Spatial analysis and source apportionment for trace gases

A strong shipping emission influence, identified by significant concentrations of SO_2_ heavily emitted from the combustion of HFO in vessels, was shown at *Site A*, when the south-westerly wind (140–250°) was the prevailing wind direction (see Supplementary Fig. 6). Mean concentrations of trace gases at *Site A*, calculated using 1-h SIFT-MS measurements under south-westerly winds were relatively larger (see Supplementary Table 2) than those observed in other studies by one to two orders of magnitude^46^. In this study, a net shipping profile of trace gases was extracted by using the difference in concentration between the downwind (*Site A*) and upwind sites (*Site B*) when south-westerly winds was observed at *Site A*. As indicated in Supplementary Fig. 6, the local shipping-source signature derived from this spatial analysis is distinguished by higher alkanes, aromatic compounds including benzene and toluene, halogenated species, inorganic species containing S and oxygenated volatile organic compounds (OVOC), such as aldehydes and ketones. These results can provide supportive explanations in identifying shipping-source signatures in the PMF analysis below.

**Fig. 4:**
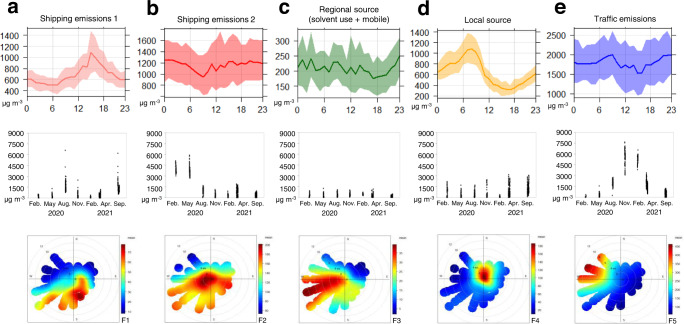
PMF source-specific diurnal/seasonal variations of gaseous pollutants at a port site and their distributions according to meteorological conditions between 2020 and 2021. **a**, **b** Shipping-related sources. **c** Regional sources including solvent use and vehicular emissions. **d** Local emissions near the study site. **e** Traffic emissions.

When this SIFT-MS dataset, consisting of 70 trace gases plus carbon monoxide (CO) was subjected to PMF, a five-factor model showed the best-fit solution in PMF (see Supplementary Fig. 7). The signature in Factor 1 was characterised by higher alkanes (nonane, decane, undecane and dodecane), having factor loadings of species ranging from 49.5 to 66.4%. This factor was also identified with some aromatic compounds (43.3% and 52.0% for styrene and naphthalene, respectively) and most halogenated species (from 24.4% for ethyl chloride to 70.2% for bromoform). In addition, high factor loading of S-containing compounds, such as carbon disulfide (58.3%) and sulfuric acid (51.7%) were observed, which may be related to the combustion of HFO. Interestingly, this PMF factor profile was highly comparable to the shipping emissions signature extracted from the above spatial analysis (see Supplementary Fig. 6). It has been reported that the higher alkanes (C > 9) and aromatics are more associated with shipping emissions, than vehicular exhausts^9^. The halogenated hydrocarbons such as chlorofluorocarbon (CFCs), tetrachloroethylene (TCE) and hexachlorobutadiene (HCBD) have been considered as indicators for aged air masses^47–50^, however the spatial analysis result in this study showed that these trace gases might be enhanced by local sources in port regions. As shown in Fig. 4a, this factor showed strong seasonality, having an enhanced contribution in summer when influenced by oceanic air masses. As well, there was a distinctive diurnal pattern with higher levels during the daytime. The PMF-derived polar plot showed that the largest concentration for trace gases at *Site A* was determined by southerly wind at with relatively low wind speed. Thus, Factor 1 was assigned to shipping emission sources.

Factor 2 was identified with relatively high loading of CO, lower alkanes, alkenes (such as 1,3-butadiene) and acetylene. Source apportionment studies by Yuan et al.^47^ have identified an aged primary factor using relatively unreactive compounds (e.g., CO and acetylene) and propane. However, the enhanced emissions for CO and hydrocarbons during manoeuvring conditions at low engine load were observed through real-time on-board measurements and was explained by incomplete combustion during transient working conditions^51^. 1,3-butadiene is known to be an important pyrolysis product of fuels^50,52,53^. As can be seen in Fig. 4b, Factor 2 has little diurnal pattern. Interestingly, a sudden decrease in the contribution from this factor was observed from August 2020, which was also seen with ambient SO_2_ at the port site (Fig. 1a). This sudden decline in factor contribution is likely to be the result of switching to a cleaner fuel. In South Korea, all ships at berth in the Emission Control Area (ECA) were required to use the lower-S fuel (<0.1%) after August 2020. As shown in the polar plot, the concentrations for trace gases tended to be highest for southerly winds at a low wind speed, and these concentrations seem to be related to shipping emissions at berth. It is likely that the implementing S restrictions on fuel oils for vessels at berth from September 2020 lead to the extraction of another factor type related to shipping emissions in PMF source apportionment analysis. While exploring three-factor to ten-factor solutions, PMF always separated out two kinds of shipping-related source types. Combined factor contribution of two PMF sources (F1 and F2) became quite comparable to the one for net shipping-source profile in the spatial analysis. Based on the literature and for reasons described, this factor was assigned to shipping emissions.

Factor 3 was explained by high factor loading of cyclic alkanes (cyclopentane, cyclohexane), isohexane (2-methylpentane) and organic aromatic compounds (benzene, toluene, xylenes + ethylbenzene and C3-alkylbenzene). It has been commonly reported that BTEX are produced through volatilisation processes (petrogenic) and they are used as source markers for solvent usages either from the industrial sector or from residential sectors in urban air^50,54–56^. Some studies have claimed that enhanced BTEX levels in urban air are related to vehicular exhaust emissions (pyrogenic)^57–59^. However, the fact that there was minor factor loading of CO mostly produced through incomplete combustion processes, may indicate that this factor is associated with non-combustion sources. Due to the different chemical reactivity of individual VOC, the ratio of toluene to benzene (*T/B*) can explain the photochemical age of the air mass^47,60^. Given the *T/B* ratio (3.9$\,{\boldsymbol{\pm }}$ 1.6 (*n* = 775)) calculated by the PMF source profile, this factor may have been influenced by mobile sources^55,61,62^. There was no distinctive seasonal/diurnal pattern for trace gases as seen in Fig. 4c. The highest concentration for gaseous species in this factor was determined by westerly winds at higher wind speed, which means this factor seems to be more associated with regional sources rather than local ones. Therefore, this factor was assigned to mixed regional sources including solvent use and mobile sources.

Factor 4 was identified with ethyl chloride of 55.9%, followed by OVOC (40.2%, 38.1% and 30.0% for methanol, ethanol and butanone respectively), 1,3-butadiene (32.1%), ethane (29.2%) and toluene (28.6%). Additionally, a meaningful contribution from CO (20.1%), a combustion source indicator, was observed in this factor. Ethyl chloride is generally used as gasoline additive, and 1,3-butadiene in urban air is known to be primarily produced as a result of incomplete combustion from internal combustion engines^52,53^. As seen in Fig. 4d, there is an apparent diurnal pattern with large amplitudes in the morning, which is slightly different from typical traffic emission patterns with bimodal distributions. It seems that trace gas levels associated with this factor are determined by local source, as the highest concentration was observed with lower wind speeds in the polar plot. In practice, this study site is surrounded by several ports and the field campaigns were conducted at one of the ports used for governmental vessels. Heavy-duty vehicles are usually parked at, or near our study site in the morning, to refuel the ships. This factor therefore seems to be explained by local emissions related to ship refuelling.

Factor 5 was identified with low-weight alkanes (propane of 55.5% and ethane of 31.1%), alkenes (propene of 74.1% and 1-pentene of 44.4%), an alkyne (acetylene of 45.8%), halogenated hydrocarbons (hexachlorobutadiene of 60.5% and tetrachloroethylene of 40.7%) and OVOC (acetaldehyde of 72.9% and formaldehyde of 56.2%, with ethanol of 41.7%). High factor loading of methyl-mercaptan (72.3%), acetonitrile (48.9%) and CO (33.9%) were also shown in this factor. A UK emission inventory for non-methane volatile organic emission compounds, reported that light alkanes and alkenes are significantly determined by combustion sources^53^. Many source apportionment studies have highlighted that light alkanes, such as butane, iso/n-pentane and hexane, are predominantly the result of traffic exhaust emissions and gasoline evaporation in urban air^50,54,57,63–65^. It has been generally reported that the significance of ethane and acetylene in urban air is attributable to traffic exhaust emissions through an incomplete combustion process^46,55^. It has also been shown that vehicular ethanol emissions on the roads, reviewed as the most abundant VOC species in London air could contribute significantly to the increased production of secondary species such as acetaldehyde^66^. The high abundance of less reactive species, such as ethane, propane, and some OVOC was explained by regional background contributions transported from remote sources, although the difficulty in separating OVOC origins between biogenic and anthropogenic has been reported^64^. As shown in Fig. 4e, trace gas concentrations associated with this factor showed a diurnal pattern with a morning peak, which is possibly influenced by anthropogenic emissions such as commuting vehicles. The polar plot showed that the concentrations tended to be highest under northwesterly winds with relatively high wind speed. In practice there are heavily trafficked main roads within 1 km to the west of the study site, carrying not only light-duty vehicles but also container trucks. A distinctive seasonality with a winter enhancement during the period with continental air masses was shown. These results and observations led us to assign factor 5 to traffic emissions, including both primary and secondary contributions.

### PMF source-specific potential for SOA formation

**Fig. 5:**
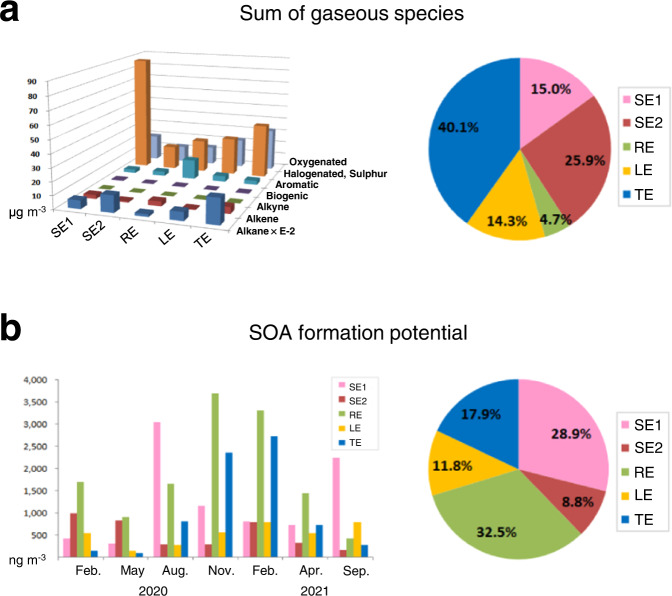
PMF source-specific contributions to gaseous air pollutants at *Site A* (SE shipping emissions, RE regional emissions including solvent use and vehicular emissions, LE local emissions, TE traffic emissions). **a** Source-specific concentrations segregated by chemical functional groups of 70 gaseous species measured by SIFT-MS. **b** The potential of secondary organic aerosol formation depending on PMF source types.

As shown in Fig. 5a, the highest mass contribution to the sum of 70 trace gases at *Site A* was observed from shipping emissions (40.9%), followed by traffic emissions (40.1%) and regional emissions including solvent use and vehicular emissions (14.3%), when using concentrations for individual trace gases as a function of PMF analysis. Interestingly, slightly different source-specific contributions to total SOA formation potentials, compared to those in total concentrations, were observed (see Fig. 5b). The highest SOA contribution was determined by shipping emissions (37.7%), followed by regional emissions (32.5%) and traffic emissions (17.9%).

It has commonly been reported that there is a strong seasonality in SOA formation potential, with significant values developing in winter at urban sites, owing to pollution dynamics in the cold season^46^. Our results are consistent with this. However, source apportionment results in this study could provide further insight into the source-dependent seasonal contribution to SOA formation. With regard to shipping sources, significantly developed potentials contributing to SOA formation were obtained in the warmer season, which may be related to dominant wind direction having a stronger influence on shipping emissions. In addition, there was a significant decrease of shipping-source contributions to SOA formation potential during the period of stricter S content regulation in 2021, compared to values in 2020. As previously mentioned the reduction of organic aerosol may be cased either by concurrent emission reductions of S and other pollutants through the combustion of cleaner fuel or by a reduction in the consumption of lubricating oils required to neutralise the acidic products.

In practice, the SOA formation potentials from shipping emissions could contribute more SOA than expected since only twelve species among 70 trace gases measured were selected when estimating SOA formation potential in this study, due to limited information on SOA yields for individual VOC species in the literature. The significance of higher alkanes (C > 10) appeared in the PMF shipping-source profile (see Supplementary Fig. 7) and intermediate VOCs (IVOC) has been highlighted in SOA formation potential^9,67,68^.

### Chemical transport model sensitivity

**Fig. 6:**
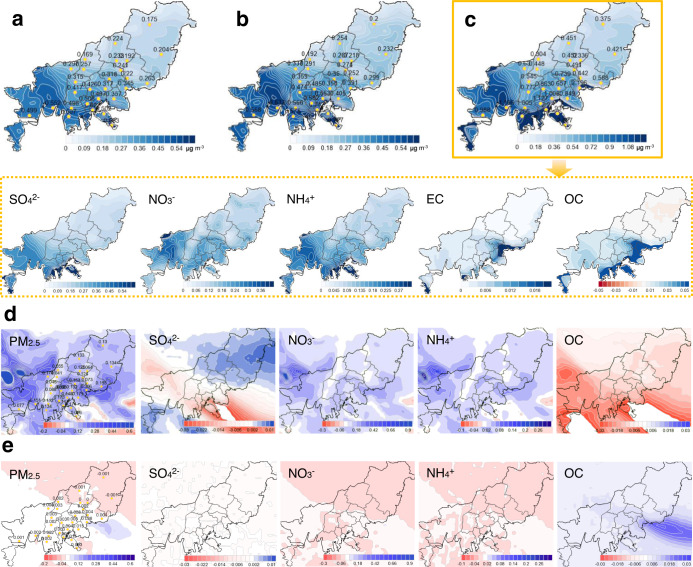
The response of surface PM_2.5_ components to shipping emission reductions on August 20, 2020 in Busan from the SMOKE-WRF/FNL-CMAQ sensitivity analysis (blue colour for mitigation and red colour for increment). The spatial impact on ambient PM_2.5_ mass over Busan by regulating S content in shipping fuel oil either (**a**) from 3.5 to 0.5% (difference between Case 1 and Case 0) or **b** from 3.5% to 0.1% (difference between Case 2 and Case 0). **c** The Influence on PM_2.5_ chemical compositions throughout shipping emissions controls including the switch to a cleaner fuel (content of S, from 3.5% to 0.1%) and mitigation of oil consumption by speed regulations in Emission Control Area (difference between Case 3 and Case 0). **d** Quantitative influence of NOx emission reduction from shipping sector on PM_2.5_ compositions in the Case 3 simulation (difference between Case 3 and Case 4). **e** Quantitative influence of VOC emission reduction from shipping sector on PM_2.5_ compositions in Case 3 simulation (difference between Case 3 and Case 5).

Without intensive air quality monitoring data, the impacts on airborne particles resulting from shipping emission controls cannot be fully understood. Therefore, in the current study, several emission-change scenarios have been simulated for Busan based on the SMOKE-WRF/FNL-CMAQ system at a 1 km resolution (see Supplementary Table 3). Surface modelling results show that the influence of shipping emissions, present dominantly along the coast, extended to inland air. As identified in Fig. 6a with shipping sector simulations, a notable reduction in surface PM_2.5_ could be achieved in both the western and southern part of Busan, when switching to lower-S (from 3.5 to 0.5%) fuel oil. It is likely that the significant mitigation of PM_2.5_ levels in southern Busan adjacent to shipping lanes is associated with reduced primary PM_2.5_ emissions as a result of changing to cleaner fuel oil. Meanwhile, the PM_2.5_ reduction farther inland to the northwest of Busan appears to be explained by a decrement in SIA, in particular ammonium sulphates (i.e., (NH_4_)_2_SO_4,_ NH_4_HSO_4_). In the western part of Busan, relatively significant levels of ambient ammonia (NH_3_) emitted from either industrial complexes or agricultural land are usually observed. This may be favourable in terms of neutralising atmospheric SO_2_ emitted from port regions and transported to inland areas. As shown in Fig. 6b, improvements in air quality could be achieved in terms of surface PM_2.5_ mass concentrations, when stricter regulation on S content for shipping fuel oils (from 3.5 to 0.1%) was simulated in Case 2. In the Case 3 scenario, additional emission controls that included an assumption of a 30% reduction in consumption of fuel oils through ship speed optimisation in ECAs, could contribute greatly to improved air quality in coastal urban air (see Fig. 6c); particularly compared to the results seen in Case 2.

When changes of PM_2.5_ constituents in the Case 3 scenario were spatially evaluated, a distinct reduction of SO_4_^2−^ was shown adjacent to the coast, while the mitigation of nitrate (NO_3_^−^) was widely developed inland. It appears that the map illustrating both SO_4_^2−^ and NO_3_^−^ together exhibits a good match to the spatial distribution of ammonium (NH_4_^+^). This may be explained by SIA formation chemistry where NH_3_ gas is a dominant precursor for neutralisation of acidic gases, such as H_2_SO_4_ and HNO_3_ through forming secondary particles. Since sulphate particle formation has priority over nitrate particle generation when reacting with NH_3_^11^, the regulation of NOx emissions from ships contributes to NO_3_^−^ reduction in inland areas, whereas SOx emission control appears to be effective in reducing SO_4_^2−^ near source (i.e., port). Due to the thermal instability of NO_3_^−^ in summer^69^, the reduction effect of NO_3_^−^ in PM_2.5_ resulting from regulation of NOx emissions may have been relatively less sensitive near source. Meanwhile, the impact of shipping emission controls on mitigation of the carbonaceous fraction in PM_2.5_ (i.e., EC and OC) was distributed relatively locally compared to the inorganic aerosol fractions. The degree of chemical reduction of EC, which is used as an indicator for primary particles produced through incomplete fuel combustion, was more concentrated near shipping lanes, compared to OC. SOA underestimation as a function of distance from source to locations downwind, is considered one of the limitations of the chemical transport model^68,70–72^ and may have resulted in underestimating the mitigation effect from reducing fuel consumption, on organic aerosols inland. A significant reduction of the carbonaceous fraction of PM_2.5_ corresponding to the shipping emissions control has been discussed earlier in PMF source apportionment results.

The impact of NOx emission reductions on PM_2.5_ constituents from decreased fuel consumption was spatially evaluated by limiting the reduction of NOx emissions from the Case 3 scenario. The difference in simulated PM_2.5_ concentrations between Cases 3 and 4 may provide an explanation for quantitative effects of the regulation on NOx emissions from shipping on particulate matters in coastal regions. As illustrated in Fig. 6d, NOx emission controls on the shipping sector provide benefits in terms of reducing PM_2.5_ concentrations, especially nitrate and ammonium components, over all of Busan. However, the opposite pattern was seen for sulphate and organic aerosol components. Levels of sulphate particles were increased in southern-east Busan, while organic aerosols were enhanced over the entire city. It has been reported that NH_3_ is more reactive with H_2_SO_4_ than HNO_3_^11^, therefore enhanced NH_3_ availability arising from NOx emission decline would appear to be an inappropriate explanation for the increment of sulphate aerosol in western Busan. On the other hand, the fact that increased hydroxyl radical (OH⋅) resulting from the reduced NOx titration can accelerate the oxidation rate of SO_2_ in secondary sulphate formation mechanism could be a possible explanation for sulphate particle enhancement near port regions. A similar explanation may be applied to the increase in organic particles. The increased atmospheric oxidants under lower NOx conditions could play a role in the development of SOA formation through oxidation of VOC.

As observed in Fig. 6e, VOC mitigation through reduced fuel oil consumption from shipping, contributed to a distinct reduction of organic aerosols in maritime air. However, little influence on other PM_2.5_ components was identified.

## Discussion

Adopting two different modelling approaches (i.e., a receptor model and numerical simulations) in the current study gives quantitative insights into the change of PM_2.5_ chemical components in coastal urban regions, corresponding to the shipping emission changes.

It has been controversial whether conventional shipping-source markers, especially V, are appropriate species for source apportionment analysis over the period covering post-S reduction^9^. However, a comparison of PMF source-specific contributions between port and urban background PM_2.5_ datasets in this study could extend our understanding of effects on PM_2.5_ chemical compositions from marine fuel regulations. In particular, our interest is in the results of lowering S content in shipping fuel oils since January 2020 (from 3.5 to 0.5–0.1%), when extensive PM_2.5_ speciation datasets covering both periods (pre-regulation and post-regulation) were applied to factor analysis. A substantial reduction in PM_2.5_ mass associated with shipping sources was observed in the coastal city of Busan over 3 years. Indeed, PM_2.5_ was reduced by 88.9% (from 8.1 μg m^−3^ in 2019 to 0.9 μg m^−3^ in 2021) and 81.3% (from 4.8 μg m^−3^ in 2019 to 0.9 μg m^−3^ in 2021) at a port and an inland urban background site, respectively. This decrease of PM_2.5_ was mostly associated with reductions in shipping-source-related SO_4_^2−^ levels. As a result, the relative contribution of major PM_2.5_ components has changed significantly in Busan over 3 years. At an urban background site, the largest contributor to PM_2.5_ was sulphate aerosols in 2019 (25.7%), but not in 2021 (16.1%). A considerable decrease of metallic components (i.e., V and Ni) in PM_2.5_, attributable to shipping sources, was also observed post-regulation. These results imply that switching to cleaner fuels in the shipping sector is likely to provide human health benefits in coastal urban regions, as also addressed in recent studies^73–75^.

Based on the WRF/FNL-CAMx/PSAT numerical modelling results appeared in Supplementary Fig. 8, only 6 days in 2020 were subjected to the CMAQ sensitivity analysis as these were when shipping sources were expected to be the largest contributor to PM_2.5_ mass over Busan. Thus, there may be limits to fully understanding the impacts on spatial/temporal change in PM_2.5_ components resulting from the regulation of shipping emissions. A high level of uncertainty in pollutant emissions has been widely considered as a limitation of interpreting the numerical model output. The difficulty in the exact estimation of the interaction between local and regional emissions from brute-force method (BFM) simulations with numerical modelling has been outlined. However, our sensitivity results demonstrated great potential for reducing PM_2.5_ bulk mass in the interior by controlling shipping emissions in port regions, particularly in summer when influenced by oceanic air masses. They also reflect that reductions in the major PM_2.5_ components may not be uniform across inland regions, owing to the complex atmospheric chemistry of PM formation during transport from near sources to receptor sites.

In parallel with the CMAQ sensitivity results obtained in this study, the application of PMF to three large datasets provided more quantitative insights into relationships between the emissions control on shipping and changes in carbonaceous fractions in PM_2.5_. The fact that there was an appreciable decrease of shipping-source contributions to both OC concentrations and to SOA formation potential during the period of post-S reduction, suggested that switching to cleaner fuels can provide considerable advantages in terms of lowering SOA in the air. Moreover, supported by PMF shipping-source signatures in this study, one may conclude that the reduction of toxic organic species such as halogenated and aromatic hydrocarbons^76^, during the period following S reduction can reduce human health risk.

The shipping-source signature of gaseous carbonaceous species might be useful in establishing detailed emission inventories for VOCs from the shipping sector, which in turn may contribute to improving representation of organic aerosols in coastal regions in CMAQ simulations. Additionally, PMF results derived in this study, suggest that traffic emissions may responsible for a substantial fraction of organic aerosols in urban air and thus need to be managed more carefully in future.

## Methods

### Observations

Information on study sites and observed data are summarised in Supplementary Table 1. Details on air quality data (CO, NO_2_, SO_2_, O_3_, PM_2.5_ and PM_10_) at AQM sites in Busan, and the chemical analysis methods for PM_2.5_ speciation at both the urban background site (Yeon-San) and the port site (Sin-Hang), are available elsewhere^77,78^. PM_2.5_ components monitored between 2019 and 2021, included automatically monitored 1-h measurements of 10 species (SO_4_^2−^, NO_3_^−^, Cl^−^, NH_4_^+^, Na^+^, Ca^2+^, Mg^2+^, K^+^, EC, OC) and manually analysed 24-hour PM_2.5_ filter samples of 20 metallic species (Al, As, Cd, Co, Cr, Cu, Fe, Li, Mn, Mo, Ni, Pb, Sb, Se, Si, Sr, Ti, Tl, V, Zn). Instrumentations and analytical methodologies vary depending on species: particulate carbonaceous species (Thermal-Optical-Transmittance, Sunset OCEC); ionic species (IC, URG 9000B Ambient Ion Monitor); metallic species (ICP-MS, PerkinElmer NexION 2000). Detailed analysis including determination of species and QA/QC has been conducted based on the procedures of PM_2.5_ chemical speciation network guidelines published by the Korean National Institute of Environmental Research^79^.

A wide range of 70 gaseous carbonaceous species were measured using a mobile laboratory equipped with a SIFT-MS near the port AQM site (Buk-Hang). Consecutive five-day field campaigns with a resolution of about 40 s were quarterly conducted at two nearby port locations (*Sites A and B*) simultaneously using two SIFT-MS from 2020 to 2021. The operating principle behind SIFT-MS is outlined elsewhere^80^. Details of the operation of the SIFT-MS are described in the guidelines published by the Korean National Institute of Environmental Research^81,82^. In this study, a TO-15 standard gas mixture was used for analysis of 70 VOCs. The linearity of the calibration curve and the quantitative reproducibility of the target compound were evaluated. More detailed information on analysis and QA/QC has been previously reported^76,83^.

### PM_2.5_ shipping emission estimates

Shipping emissions were calculated using the bottom-up approaches, where pollutants’ emissions are estimated for the individual ships^4^. Estimations of fuel consumption from shipping sector were made for the period between 2019 and 2021 using an automatic port management information system (AIS) information to calculate PM_2.5_ emissions^84^, because the national emission estimates from 2020 were not available. Details on estimating air pollutant emissions attributable to shipping have been described elsewhere^85–87^.

The equation used to calculate primary PM_2.5_ emissions was:

1 $${\rm{E}}_{ij}=({\rm{Fh}}{w}_{j}+{\rm{Fm}}{w}_{j})\times {{\rm{EF}}}_{i}\times {10}^{-{{3}}}$$

where E_*ij*_ is the *i*th trace species emissions in the *j*th vessel (tonnes), Fh*w*_*j*_ is the *w*_th_ gross tonnage *j*th vessel fuel consumptions during hoteling (tonnes), Fm*w*_*j*_ is the *w*_th_ gross tonnage *j*th vessel fuel consumptions during manoeuvring (tonnes) and EF_*i*_ is *i*th trace species emission factor in shipping fuel oils (kg tonnes^−1^), 5.6, 79.3, 20 S, 2.7 for PM_2.5_, NOx, SO_2_ and VOC, respectively.

2 $${\rm{Fh}}{w}_{j}={\rm{SFOC}}\times {\rm{t}}\times 0.2$$

3 $${\rm{Fm}}{w}_{j}={\rm{D}}\times 2\times {{\rm{M}}}^{-1}\times \rho$$

where SFOC is the fuel oil consumption coefficient at maximum speed of *w*_th_ gross tonnage *j*th vessel calculated as *w*_th_ gross tonnage × 10^-3^ + 16.263 (tonnes day^−1^), *t* is the time (days) spent in hoteling phase for *w*_th_ gross tonnage *j*th vessel and 0.2 is the assumed ratio of fuel consumption between in hoteling phase and at maximum speed. D is the manoeuvring distance (km), 2 is the arrival and departure, M is the *w*_th_ gross tonnage *j*th vessel fuel efficiency (km k*l*^−1^) and *ρ* is the specific gravity of shipping fuel oils (0.9593 tonnes k*l*^−1^).

4 $${{\rm{E}}}_{j}({2}nd)=0.345\times {{\rm{E}}}_{{\rm{SO}}2j}+0.079\times {{\rm{E}}}_{{\rm{NO}}xj}+0.024\times {{\rm{E}}}_{{\rm{VOC}}j}$$

where E_*j*_ *(2nd)* is the estimated secondary PM_2.5_ emissions (tonnes), and E_SO2*j*_, E_NOx*j*_, and E_VOC*j*_ are the estimated emissions, respectively, for SO_2_, NOx and VOC, derived from the fuel consumption method in Eqs. (1–3).

### PMF source apportionment

In this study, a PMF modelling approach was applied to identify shipping-source-related PM_2.5_ and trace gases, and to quantify source-specific contributions either in urban air or in port air over Busan. The importance of using high-quality input data or assessing the source identification performances of receptor models was emphasised to ensure reliable air quality evaluation^88^. Dataset preparation and modelling solution optimisation procedures throughout the source apportionment analysis using PMF have been described elsewhere^89–91^.

Two 24-h PM_2.5_ speciation datasets (port site and urban background site), which consisted of measurements taken before regulations (2019) and during the period of post-S reduction in shipping fuel oils (2020–2021), were prepared. A 774 × 31 matrix (sample number × species) PM_2.5_ dataset for port site and a 751 × 31 matrix PM_2.5_ dataset for urban background site were introduced into the PMF 5.0. With regard to SIFT-MS measurements at *Site A*, a 997 × 71 matrix dataset was prepared by including hourly measurements of 70 gaseous species and CO, where a typical tracer of primary combustion sources, i.e., CO could separate out the two different source types i.e., combustion-related and volatilisation-associated^64^. The PMF input data files consist of measurements (C) and their uncertainties (U). In this work, an uncertainty of individual species for three datasets was estimated using concentrations and method detection limit (MDL)^92^, as revealed in Eq. (5).

5 $${{\rm{U}}}_{ij}={\rm{k}}\times {{\rm{C}}}_{ij}+{\rm{MDL}}/3$$

where U_*ij*_ is the uncertainty of species *j* in sample *i*, k is the analytical error fraction (0.1 when C_*ij*_ > MDL; 0.2 when C_*ij*_ ≦ MDL), and MDL is the detection limit determined by means of repeated measurements.

The PMF solutions for three datasets were respectively evaluated by varying the parameters such as number of factors (3–10), extra modelling uncertainty (0–30%), and species-specific uncertainty via selection of the category of each compounds (“Strong”, “Weak”, or “Bad”). Fpeak analysis was performed by changing Fpeak strength from −1 to 1 in steps of 0.4, and the Fpeak value was set to zero. The correlation between the observed and modelled concentrations for individual species was examined. Bootstrapping was implemented to assess the stability of PMF solutions. In addition, rotational ambiguity and effects from random errors were assessed through DISP and BS-DISP analysis. Finally, optimised solutions were determined based on literature reviews for source markers, recognised local sources and source-specific contributions as a function of meteorological data. With regards to SIFT-MS dataset, two types of complementary approaches (inclusion of CO, and spatial analysis) were adopted to evaluate the adequacy of shipping-source identification for PMF factor profiles of trace gases, as limited information on source signatures for a wide range of carbonaceous species associated with shipping emissions has been reported.

### Spatial cross-sectional analysis and SOA formation potential

The extraction of specific local source profiles based on cross-sectional analysis using the different concentrations between upwind and downwind sites is also outlined in the previous study^89^. Prior to the cross-sectional analysis, 5-min mean concentrations of carbonaceous organic gaseous species were calculated using SIFT-MS measurements with a resolution of 40 s at *Sites A and B*. The 1-hour mean concentrations were produced when 5-min mean concentrations were concurrently available at both sites, which were used either for spatial analysis or for PMF analysis. A dominant wind direction having strong shipping emission influence at *Site A* could be identified by using a polar plot of SO_2_ (retrieved from the port site) heavily emitted through HFO combustion from vessels in port regions (see Supplementary Fig. 6). Therefore, during the ship-plume-influenced times, the enhanced hourly level of carbonaceous organic species at downwind sites (i.e., *Site A*) transported over port regions from the upwind site (i.e., *Site B*) could be the result of shipping emissions. The net contribution profile at *Site A* was obtained by calculating (C_A_ - C_B_) / C_A_ × 100. And this source signature could provide a supportive explanation in the identification of PMF factor profile for gaseous pollutants.

Based on the PMF source profiles for trace gas species, the source-dependent potential for the formation of SOA was estimated as below.

6 $${{\rm{SOA}}}_{i}={{\rm{VOC}}}_{i}\times {\rm{SOA}}\,{{\rm{yield}}}_{i}$$

where SOA*i* is the potential of secondary organic aerosol formation of individual VOC species (ng m^−3^), VOC_*i*_ is the concentration of *i* species (ppbv), and SOA yield_*i*_ is the organic aerosol production yield derived from the experimental photochemistry of *i* species in other publications (μg m^−3^ ppm^−1^). In this study, available information for the SOA yield of twelve VOC species (heptane, iso/n-octane, nonane, decane, undecane, cyclohexane, acetylene, isoprene, benzene, toluene, xylenes/ethylbenzene, styrene) was used for calculating SOA formation potential^93–95^.

### CMAQ sensitivity

The effect on air quality from abatement policies for shipping emissions, such as switches to the lower-S fuels and reducing sailing speed, need to be assessed spatially. To achieve this, a chemical transport modelling (CMAQ) approach was adopted to get quantitative insights into the relationship between changes of precursor emissions (i.e., SO_2_, NO_x_, NH_3_, VOC), resulting from implementing regulations on shipping, and the PM_2.5_ chemical compositions. A detailed description of high-resolution CMAQ simulations over Busan and modelling evaluations are available elsewhere^96^. Briefly, the Weather Research Forecasting model with NCEP Global Final Analysis (WRF/FNL) were applied to CMAQ simulation of a 1 km resolution over Busan. The anthropogenic emissions used in the CMAQ simulations were derived from the 1 × 1 km spatial resolution national emission estimates. As shown in Supplementary Fig. 8, the study period (from August 20, 2020 to August 26, 2020) for the CMAQ sensitivity simulations was selected based on the source apportionment results of WRF/FNL-CAMx with Particulate matter Source Apportionment Technology (PSAT) tool, in which the daily mean PM_2.5_ level in Busan is predominantly determined by local emissions and of which, shipping source is the most significant contributor. A 5-day spin-up period was used to prevent initial conditions having an effect on the simulated PM_2.5_ of the study period^97^.

Several emission control scenarios were performed using CMAQ modelling and are summarised in Supplementary Table 3. A reference run including all emissions (Case 0) and other runs without shipping emissions (Cases 1–3) were simulated. Then, the difference in PM_2.5_ surface levels at a 1 km resolution between the reference run and the other runs was calculated, tracking the effect of shipping emission controls. Moreover, sensitivity of PM_2.5_ composition changes in the Case 3 scenario, derived either from the NOx reduction (Case 4) or from the VOC reduction (Case 5), was assessed.

## Supplementary information

Supplementary Information for Impact of shipping emissions regulation: Urban aerosol composition changes revealed by receptor and numerical modelling

## Data Availability

Data on shipping activities used in this paper can be found at https://new.portmis.go.kr. PM_2.5_ chemically-speciated data and AQM data can be downloaded from the website of the Busan Metropolitan City Institute of Health and Environment (http://heis.busan.go.kr/). The SIFT-MS trace gas datasets used in this study are available upon reasonable request by contacting the corresponding author (jjena77@korea.kr).

## Code availability

The codes used for analyses in this study are available on request from the authors.

## References

[1] Donateo A (2014). Contribution of harbour activities and ship traffic to PM2.5, particle number concentrations and PAHs in a port city of the Mediterranean Sea (Italy). Environ. Sci. Pollut. Res..

[2] Eyring V, Köhler H, Van Aardenne J, Lauer A (2005). Emissions from international shipping: 1. The last 50 years. J. Geophys. Res. Atmos..

[3] Endresen Ø (2003). Emission from international sea transportation and environmental impact. J. Geophys. Res. Atmos..

[4] Merico E (2021). Shipping and air quality in Italian port cities: state-of-the-art analysis of available results of estimated impacts. Atmosphere.

[5] European Environment Agency. *Aviation and Shipping—IMPACTS on Europe’s Environment*. (EEA Report, No 22/2017, 2017).

[6] Agrawal H (2009). Primary particulate matter from ocean-going engines in the Southern California Air Basin. Environ. Sci. Technol..

[7] Chen G (2005). An investigation of the chemistry of ship emission plumes during ITCT 2002. J. Geophys. Res. Atmos..

[8] Contini D (2011). The direct influence of ship traffic on atmospheric PM2.5, PM10 and PAH in Venice. J. Environ. Manag..

[9] Xiao Q (2018). Characteristics of marine shipping emissions at berth: profiles for particulate matter and volatile organic compounds. Atmos. Chem. Phys..

[10] Ault AP, Moore MJ, Furutani H, Prather KA (2009). Impact of emissions from the Los Angeles port region on San Diego air quality during regional transport events. Environ. Sci. Technol..

[11] McMurry, P. H., Shepherd, M. F. & Vickery, J. S. *Particulate Matter Science for Policy Makers: A NARSTO Assessment* (Cambridge University Press, 2004).

[12] Pope CA, Dockery DW (2006). Health effects of fine particulate air pollution: lines that connect. J. Air Waste Manag. Assoc..

[13] Sacks, J. et. al. *Integrated Science Assessment for Particulate Matter*. (US Environmental Protection Agency, EPA/600/R-19/188, 2019). 36630543

[14] World Health Organization. Health aspects of air pollution with particulate matter, ozone and nitrogen dioxide: report on a WHO working group, Bonn, Germany 13–15 January 2003. Copenhagen: WHO Regional Office for Europe (2003).

[15] Andersson C, Bergström R, Johansson C (2009). Population exposure and mortality due to regional background PM in Europe – Long-term simulations of source region and shipping contributions. Atmos. Environ..

[16] Viana M (2020). Estimated health impacts from maritime transport in the Mediterranean region and benefits from the use of cleaner fuels. Environ. Int..

[17] Corbett JJ (2007). Mortality from ship emissions: a global assessment. Environ. Sci. Technol..

[18] Viana M (2009). Chemical tracers of particulate emissions from commercial shipping. Environ. Sci. Technol..

[19] Henschel, S. & Chan, G. *Health risks of air pollution in Europe – HRAPIE project: new emerging risks to health from air pollution – results from the survey of experts*. (World Health Organization, Regional office for Europe, 2013).

[20] Song S-K (2022). Impact of International Maritime Organization 2020 sulfur content regulations on port air quality at international hub port. J. Clean. Prod..

[21] Lin H (2018). Shipping pollution emission associated with increased cardiovascular mortality: a time series study in Guangzhou, China. Environ. Pollut..

[22] Cao J, Xu H, Xu Q, Chen B, Kan H (2012). Fine particulate matter constituents and cardiopulmonary mortality in a heavily polluted Chinese city. Environ. Health Perspect..

[23] Campen MJ (2001). Cardiovascular and thermoregulatory effects of inhaled PM-associated transition metals: a potential interaction between nickel and vanadium sulfate. Toxicol. Sci..

[24] Healy RM (2009). Characterisation of single particles from in-port ship emissions. Atmos. Environ..

[25] Streibel T (2017). Aerosol emissions of a ship diesel engine operated with diesel fuel or heavy fuel oil. Environ. Sci. Pollut. Res..

[26] Merico E (2016). Influence of in-port ships emissions to gaseous atmospheric pollutants and to particulate matter of different sizes in a Mediterranean harbour in Italy. Atmos. Environ..

[27] Merico E (2017). Atmospheric impact of ship traffic in four Adriatic-Ionian port-cities: comparison and harmonization of different approaches. Transport. Res. Part D: Transp. Environ..

[28] Xu L (2018). Source identification of PM2. 5 at a port and an adjacent urban site in a coastal city of China: impact of ship emissions and port activities. Sci. Total Environ..

[29] Scerri MM (2018). Estimation of the contributions of the sources driving PM2. 5 levels in a Central Mediterranean coastal town. Chemosphere.

[30] Bove M (2014). An integrated PM2. 5 source apportionment study: positive matrix factorisation vs. the chemical transport model CAMx. Atmos. Environ..

[31] Verschuur J, Koks EE, Hall JW (2021). Global economic impacts of COVID-19 lockdown measures stand out in high-frequency shipping data. PLoS ONE.

[32] Nightingale, L. *Lloyd’s list one hundred ports 2021*. (PORT of vancouver, 2021).

[33] National Air Emission Inventory and Research Center. National air pollutants emission 2019. https://www.air.go.kr/en-main (2022).

[34] Dabek-Zlotorzynska E (2011). Canadian National Air Pollution Surveillance (NAPS) PM2.5 speciation program: Methodology and PM2.5 chemical composition for the years 2003–2008. Atmos. Environ..

[35] Zhang X, Murakami T, Wang J, Aikawa M (2021). Sources, species and secondary formation of atmospheric aerosols and gaseous precursors in the suburb of Kitakyushu, Japan. Sci. Total Environ..

[36] Robock A (2000). Volcanic eruptions and climate. Rev. Geophys..

[37] Cesari D (2014). Source apportionment of PM2. 5 in the harbour–industrial area of Brindisi (Italy): identification and estimation of the contribution of in-port ship emissions. Sci. Total Environ..

[38] Zhang X (2019). Changes in the SO2 level and PM2. 5 components in Shanghai driven by implementing the ship emission control policy. Environ. Sci. Technol..

[39] Zhao J (2021). Trace elements from ocean‐going vessels in East Asia: vanadium and nickel emissions and their impacts on air quality. J. Geophys. Res. Atmos..

[40] Manousakas M (2017). Assessment of PM2.5 sources and their corresponding level of uncertainty in a coastal urban area using EPA PMF 5.0 enhanced diagnostics. Sci. Total Environ..

[41] Becagli S (2012). Evidence for heavy fuel oil combustion aerosols from chemical analyses at the island of Lampedusa: a possible large role of ships emissions in the Mediterranean. Atmos. Chem. Phys..

[42] Gentner DR (2012). Elucidating secondary organic aerosol from diesel and gasoline vehicles through detailed characterization of organic carbon emissions. Proc. Natl Acad. Sci. USA.

[43] Mamoudou I, Zhang F, Chen Q, Wang P, Chen Y (2018). Characteristics of PM2.5 from ship emissions and their impacts on the ambient air: a case study in Yangshan Harbor, Shanghai. Sci. Total Environ..

[44] Liu Z (2017). Influence of ship emissions on urban air quality: a comprehensive study using highly time-resolved online measurements and numerical simulation in Shanghai. Environ. Sci. Technol..

[45] Diesch J-M, Drewnick F, Klimach T, Borrmann S (2013). Investigation of gaseous and particulate emissions from various marine vessel types measured on the banks of the Elbe in Northern Germany. Atmos. Chem. Phys..

[46] Li Q (2020). An investigation into the role of VOCs in SOA and ozone production in Beijing, China. Sci. Total Environ..

[47] Yuan B (2012). Volatile organic compounds (VOCs) in urban air: how chemistry affects the interpretation of positive matrix factorization (PMF) analysis. J. Geophys. Res. Atmos..

[48] Brown SG, Frankel A, Hafner HR (2007). Source apportionment of VOCs in the Los Angeles area using positive matrix factorization. Atmos. Environ..

[49] Chen W (2014). Understanding primary and secondary sources of ambient carbonyl compounds in Beijing using the PMF model. Atmos. Chem. Phys..

[50] Fukusaki Y (2021). Source region identification and source apportionment of volatile organic compounds in the Tokyo Bay coastal area, Japan. Atmos. Environ.: X.

[51] Chu-Van T (2019). A comparison of particulate matter and gaseous emission factors from two large cargo vessels during manoeuvring conditions. Energy Rep..

[52] Curren KC, Dann TF, Wang DK (2006). Ambient air 1,3-butadiene concentrations in Canada (1995–2003): seasonal, day of week variations, trends, and source influences. Atmos. Environ..

[53] Lewis, A., Carslaw, D. & Moller, S. J. *Air quality expert group, Non-methane volatile organic compounds in the UK*. (DEFRA, Research report, 2020).

[54] Cai C, Geng F, Tie X, Yu Q, An J (2010). Characteristics and source apportionment of VOCs measured in Shanghai, China. Atmos. Environ..

[55] Thera BTP (2019). Composition and variability of gaseous organic pollution in the port megacity of Istanbul: source attribution, emission ratios, and inventory evaluation. Atmos. Chem. Phys..

[56] Liu B (2016). Characterization and source apportionment of volatile organic compounds based on 1-year of observational data in Tianjin, China. Environ. Pollut..

[57] Baudic A (2016). Seasonal variability and source apportionment of volatile organic compounds (VOCs) in the Paris megacity (France). Atmos. Chem. Phys..

[58] Gaimoz C (2011). Volatile organic compounds sources in Paris in spring 2007. Part II: source apportionment using positive matrix factorisation. Environ. Chem..

[59] Tsai J-H, Lu Y-T, Chung I, Chiang H-L (2020). Traffic-related airborne VOC profiles variation on road sites and residential area within a microscale in urban area in Southern Taiwan. Atmosphere.

[60] Dongarrà G, Manno E, Varrica D, Lombardo M, Vultaggio M (2010). Study on ambient concentrations of PM10, PM10–2.5, PM2. 5 and gaseous pollutants. Trace elements and chemical speciation of atmospheric particulates. Atmos. Environ..

[61] Miri M (2016). Investigation of outdoor BTEX: Concentration, variations, sources, spatial distribution, and risk assessment. Chemosphere.

[62] Su Y-C (2019). Source apportionment of volatile organic compounds (VOCs) by Positive Matrix Factorization (PMF) supported by model simulation and source markers-using petrochemical emissions as a showcase. Environ. Pollut..

[63] Song S-K (2019). Source apportionment of VOCs and their impact on air quality and health in the megacity of Seoul. Environ. Pollut..

[64] Debevec C (2017). Origin and variability in volatile organic compounds observed at an Eastern Mediterranean background site (Cyprus). Atmos. Chem. Phys..

[65] Fan M-Y (2021). Source apportionments of atmospheric volatile organic compounds in Nanjing, China during high ozone pollution season. Chemosphere.

[66] Dunmore RE (2016). Atmospheric ethanol in London and the potential impacts of future fuel formulations. Faraday Discuss..

[67] Yuan B (2013). VOC emissions, evolutions and contributions to SOA formation at a receptor site in eastern China. Atmos. Chem. Phys..

[68] Ait-Helal W (2014). Volatile and intermediate volatility organic compounds in suburban Paris: variability, origin and importance for SOA formation. Atmos. Chem. Phys..

[69] Cesari D (2018). Seasonal variability of PM2.5 and PM10 composition and sources in an urban background site in Southern Italy. Sci. Total Environ..

[70] Wyat Appel K, Bhave PV, Gilliland AB, Sarwar G, Roselle SJ (2008). Evaluation of the community multiscale air quality (CMAQ) model version 4.5: Sensitivities impacting model performance; Part II—particulate matter. Atmos. Environ..

[71] Tesche TW (2006). CMAQ/CAMx annual 2002 performance evaluation over the eastern US. Atmos. Environ..

[72] GROUP, A. Fine Particulate Matter (PM 2.5) in the United Kingdom. *Department for Environment, Food and Rural Affairs, London* (2012).

[73] Broome RA (2016). The mortality effect of ship-related fine particulate matter in the Sydney greater metropolitan region of NSW, Australia. Environ. Int..

[74] Viana M (2015). Environmental and health benefits from designating the Marmara sea and the Turkish Straits as an emission control area (ECA). Environ. Sci. Technol..

[75] Sofiev M (2018). Cleaner fuels for ships provide public health benefits with climate tradeoffs. Nat. Commun..

[76] Son HD, An JG, Ha SY, Kim GB, Yim UH (2018). Development of real-time and simultaneous quantification of volatile organic compounds in ambient with SIFT-MS (selected ion flow tube-mass spectrometry). J. Korean Soc. Atmos. Environ..

[77] Jang E, Do W, Park G, Kim M, Yoo E (2017). Spatial and temporal variation of urban air pollutants and their concentrations in relation to meteorological conditions at four sites in Busan, South Korea. Atmos. Pollut. Res..

[78] De Foy B, Heo J, Kang J-Y, Kim H, Schauer JJ (2021). Source attribution of air pollution using a generalized additive model and particle trajectory clusters. Sci. Total Environ..

[79] Hong, Y. et al. *PM2.5 chemical speciation network guidelines (in Korean)*. (National Institute of Environmental Research, NIER-GP2014-102, 2014).

[80] Smith D, Španěl P (2005). Selected ion flow tube mass spectrometry (SIFT-MS) for on-line trace gas analysis. Mass Spectrom. Rev..

[81] Ryu, S. et al. *SIFT-MS Operating Guidelines (in Korean)*. (National Institute of Environmental Research, NIER-GP2020-016, 2020).

[82] Seo, Y. et al. *SIFT-MS Operating Guidelines (in Korean).* (National Institute of Environmental Research, NIER-GP2021-037, 2021).

[83] Hwang K, An J, Lee S, Choi W, Yim U (2020). A Study on the ozone formation potential of volatile organic compounds in Busan using SIFT-MS. J. Korean Soc. Atmos. Environ..

[84] Ministry of Oceans and Fisheries. PORT-MIS. https://new.portmis.go.kr/portmis (2022).

[85] Busan Metropolitcan City. Inventory report of particulate matter emissions in Busan (in Korean). https://www.busan.go.kr/index (2019).

[86] An, Y., Yuk, G. & Kim, J. *Improvement in the estimation of air pollution emissions from ships (in Korean).* (Korea Maritime Institute, Research report 2017-35, 2017).

[87] National Air Emission Inventory and Research Center. Informative national emission inventory report (in Korean). https://www.air.go.kr/en-main (2021).

[88] Belis CA (2020). Evaluation of receptor and chemical transport models for PM10 source apportionment. Atmos. Environ.: X.

[89] Jang, E., Alam, M. S. & Harrison, R. M. Source apportionment of polycyclic aromatic hydrocarbons in urban air using positive matrix factorization and spatial distribution analysis. *Atmos. Environ.* **79**, 271–285 (2013).

[90] Jang E, Jeong T, Yoon N, Jeong S (2020). Source apportionment of airborne PCDD/F at industrial and urban sites in Busan, South Korea. Chemosphere.

[91] Norris, G., Duvall, R., Brown, S. & Bai, S. *EPA positive matrix factorization (PMF) 5.0 fundamentals and user guide.* (US Environmental Protection Agency, EPA/600/R-14/108, 2014)*.*

[92] Tauler R (2009). Comparison of the results obtained by four receptor modelling methods in aerosol source apportionment studies. Atmos. Environ..

[93] Pandis SN, Harley RA, Cass GR, Seinfeld JH (1992). Secondary organic aerosol formation and transport. Atmos. Environ. Part A. Gen. Top..

[94] Na K, Song C, Cocker DR (2006). Formation of secondary organic aerosol from the reaction of styrene with ozone in the presence and absence of ammonia and water. Atmos. Environ..

[95] Shin H, Kim J, Lee S, Kim Y (2013). Evaluation of the optimum volatile organic compounds control strategy considering the formation of ozone and secondary organic aerosol in Seoul, Korea. Environ. Sci. Pollut. Res..

[96] Jang E, Kim M, Do W, Park G, Yoo E (2022). Real-time estimation of PM2. 5 concentrations at high spatial resolution in Busan by fusing observational data with chemical transport model outputs. Atmos. Pollut. Res..

[97] Karl M (2019). Effects of ship emissions on air quality in the Baltic Sea region simulated with three different chemistry transport models. Atmos. Chem. Phys..

