# Supplementary material for: Impact of shipping emissions regulation on urban aerosol composition changes revealed by receptor and numerical modelling
Source: NPJ Clim Atmos Sci. 2023 May 29;6(1):52. doi: 10.1038/s41612-023-00364-9 (PMC10226717; doi:10.1038/s41612-023-00364-9)
Supplement: Supplementary file 1 — Supplementary Information for Impact of shipping emissions regulation: Urban aerosol composition changes revealed by receptor and numerical modelling [file 41612_2023_364_MOESM1_ESM.pdf]

Supplementary Information for

# Impact of shipping emissions regulation: Urban aerosol composition changes revealed by receptor and numerical modelling

Eunhwa Jang[1*], Seongwoo Choi[1], Eunchul Yoo[1], Sangmin Hyun[2] and Joongeon An[3]

[1] Busan Metropolitan City Institute of Health and Environment, 120, Hambakbong-ro, 140beon-gil, Buk-gu, Busan, 46616, Republic of Korea

[2] Marine Environmental Research Center, Korea Institute of Ocean Science and Technology, 385, Haeyang-ro, Yeongdo-gu, Busan, 49111, Republic of Korea

[3] Risk Assessment Research Center, Korea Institute of Ocean Science and Technology, Geoje, 53201, Republic of Korea

[*]Corresponding authors: jjena77@korea.kr (Eunhwa Jang)

This PDF file includes:

Supplementary Tables 1 to 3

Supplementary Figures 1 to 8

## Supplementary Tables

**Supplementary Table 1. Information on study sites and observed data.**

| Site Information | | | | Data information | | |
|---|---|---|---|---|---|---|
| Site name | Longitude | Latitude | Environment type | Measurements | | Temporal resolution |
| Buk-Hang | 129.055280 | 35.122570 | Port | AQM | 6 species | hourly |
| Site A | 129.055080 | 35.125586 | | SIFT-MS | 70 trace gases | every 40 sec. |
| Site B | 129.048766 | 35.114855 | | SIFT-MS | 70 trace gases | every 40 sec. |
| Sin-Hang | 128.834390 | 35.074690 | | AQM | 6 species | hourly |
| | | | | $PM_{2.5}$ speciation | 8 ionic and 2 carbonaceous species | hourly |
| | | | | | 23 metallic species | daily |
| Yeon-San | 129.074590 | 35.181150 | Urban background | AQM | 6 species | hourly |
| | | | | $PM_{2.5}$ speciation | 8 ionic and 2 carbonaceous species | hourly |
| | | | | | 23 metallic species | daily |

**Supplementary Table 2. Hourly mean concentrations (μg $m^{-3}$) of trace gases measured by a mobile laboratory equipped with selected ion flow tube mass spectrometery (SIFT-MS) at near port site (*Site A*) (n = 997).**

| Group | Species | Concentrations (Mean ± S.D.) | Group | Species | Concentrations (Mean ± S.D.) |
|---|---|---|---|---|---|
| alkane | Methane | 2874.10 ± 714.64 | halogenated | Vinyl chloride; 1,2-dichloroethane | 1.34 ± 0.79 |
| | Ethane; Ethene | 25.06 ± 15.56 | | 1,1,1-trichloroethane | 3.96 ± 2.31 |
| | Propane | 1036.62 ± 633.37 | | 1,1,2-trichloroethane | 12.03 ± 7.36 |
| | Butane | 39.77 ± 15.54 | | Ethyl chloride; 1,1-dichloroethane | 8.87 ± 7.37 |
| | Pentane (iso+n) | 87.03 ± 62.65 | | Dichloropropene | 1.07 ± 0.95 |
| | Hexane | 23.53 ± 17.68 | | 1,2-dichloropropane | 10.51 ± 4.86 |
| | Heptane | 2.69 ± 1.82 | | 1,2,4-trichlorobenzene | 1.41 ± 1.85 |
| | Octane (iso+n) | 9.79 ± 7.68 | | 1,4-dichlorobenzene | 0.55 ± 0.46 |
| | Nonane | 1.58 ± 1.40 | | Chlorobenzene | 2.32 ± 1.61 |
| | Decane | 2.72 ± 2.15 | | 1-chlorotoluene | 2.06 ± 1.26 |
| | Undecane | 3.34 ± 3.05 | | Freon11; Freon113; Freon114 | 5.65 ± 4.12 |
| | Dodecane | 1.73 ± 3.20 | | Freon12; Freon114 | 17.39 ± 12.02 |
| | Cyclopentane | 6.27 ± 4.39 | | Carbon tetrachloride | 4.69 ± 3.85 |
| | Cyclohexane | 11.02 ± 9.36 | | Tetrachloroethylene | 3.51 ± 2.87 |
| | 2-methylpentane | 3.45 ± 3.28 | others | Hexachlorobutadiene | 0.70 ± 2.82 |
| alkene | Propene | 1.69 ± 1.49 | | Carbonyl sulfide | 3.44 ± 1.87 |
| | 2-butene | 1.66 ± 1.15 | | Dimethyl sulfide | 0.80 ± 0.46 |
| | 1-pentene | 4.59 ± 3.21 | | Carbon disulfide | 8.18 ± 6.03 |
| | 1-hexene | 3.48 ± 2.26 | | Sulfuric acid | 29.34 ± 16.43 |
| | 1,3-butadiene | 0.26 ± 0.47 | | Methyl mercaptan | 0.60 ± 0.60 |
| alkyne | Acetylene | 0.25 ± 0.52 | | Acetonitrile | 0.79 ± 0.72 |
| biogenic | Isoprene | 0.26 ± 0.18 | | Ammonia | 8.93 ± 8.72 |
| | Myrcene | 1.52 ± 1.54 | oxygenated | Methanol | 9.93 ± 6.29 |
| aromatic | Benzene; Dimethyl sulfoxide | 2.55 ± 4.19 | | Ethanol; Formic acid | 16.96 ± 15.68 |
| | Toluene | 8.08 ± 8.29 | | Formaldehyde | 9.23 ± 3.94 |
| | Xylenes + Ethylbenzene | 7.80 ± 13.33 | | Acetaldehyde | 7.67 ± 5.88 |
| | Styrene | 0.76 ± 0.57 | | Propanal; Propylene oxide | 5.24 ± 2.84 |
| | C3-alkylbenzene | 4.10 ± 3.97 | | Acetone | 8.62 ± 3.85 |
| | Naphthalene | 1.37 ± 1.05 | | Butanone | 3.49 ± 3.38 |
| halogenated | Bromoform | 0.89 ± 1.16 | | Methyl isobutyl ketone | 1.56 ± 1.09 |
| | Chloroform; Dichloromethane; Tetrachloroethane | 20.61 ± 13.94 | | Tetrahydrofuran | 6.32 ± 5.30 |
| | Methyl bromide | 11.12 ± 7.49 | | Methyl tert-butyl ether | 8.54 ± 6.09 |
| | 1,2-dibromoethane | 4.54 ± 3.61 | | Vinyl acetate | 2.94 ± 2.39 |
| | Trichloroethylene | 1.24 ± 1.14 | | Acetic acid | 6.45 ± 3.89 |
| | Dichloroethene (1,1 and 1,2) | 10.32 ± 6.33 | | Butanoic acid | 1.49 ± 1.12 |

**Supplementary Table 3. CMAQ scenarios for emission control in the shipping sector.**

| Scenarios | Emission controlling design |
|---|---|
| Case 0 | Original baseline scenario |
| Case 1 | $SO_2$ emissions reduction by 85.7% from switch to low-sulphur fuels (3.5% to 0.5%) |
| Case 2 | $SO_2$ emissions reduction by 97.1% from switch to low-sulphur fuels (3.5% to 0.1%) |
| Case 3 | $SO_2$ emissions reduction by 97.1% and shipping emission reduction of 6 species (SOx, NOx, VOC, $NH_3$, CO and primary $PM_{2.5}$) by 30% |
| Case 4 | Exception of NOx emission reduction from scenario Case 3 |
| Case 5 | Exception of VOC emission reduction from scenario Case 3 |

## Supplementary Figures

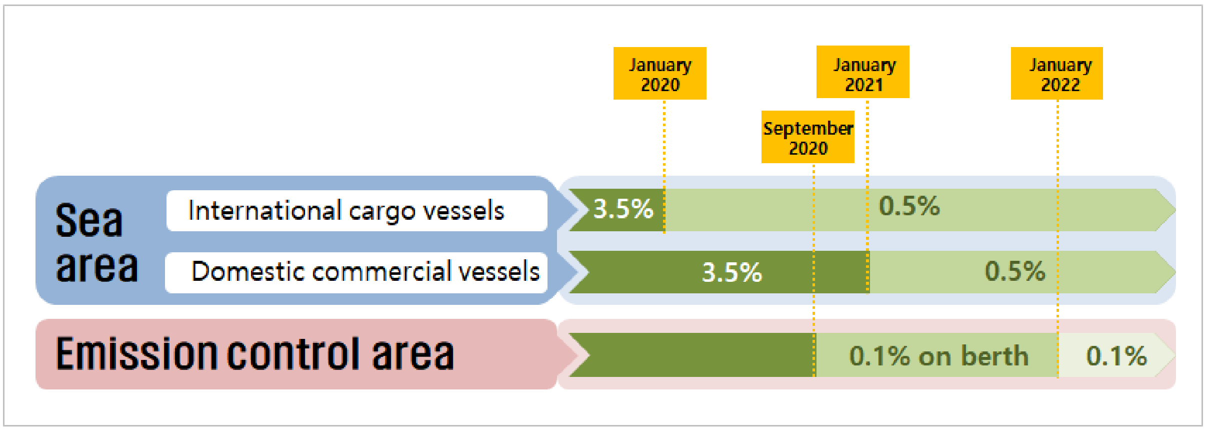


**Supplementary Figure 1. History of sulphur (S) content regulations on shipping fuel oils in Busan**.

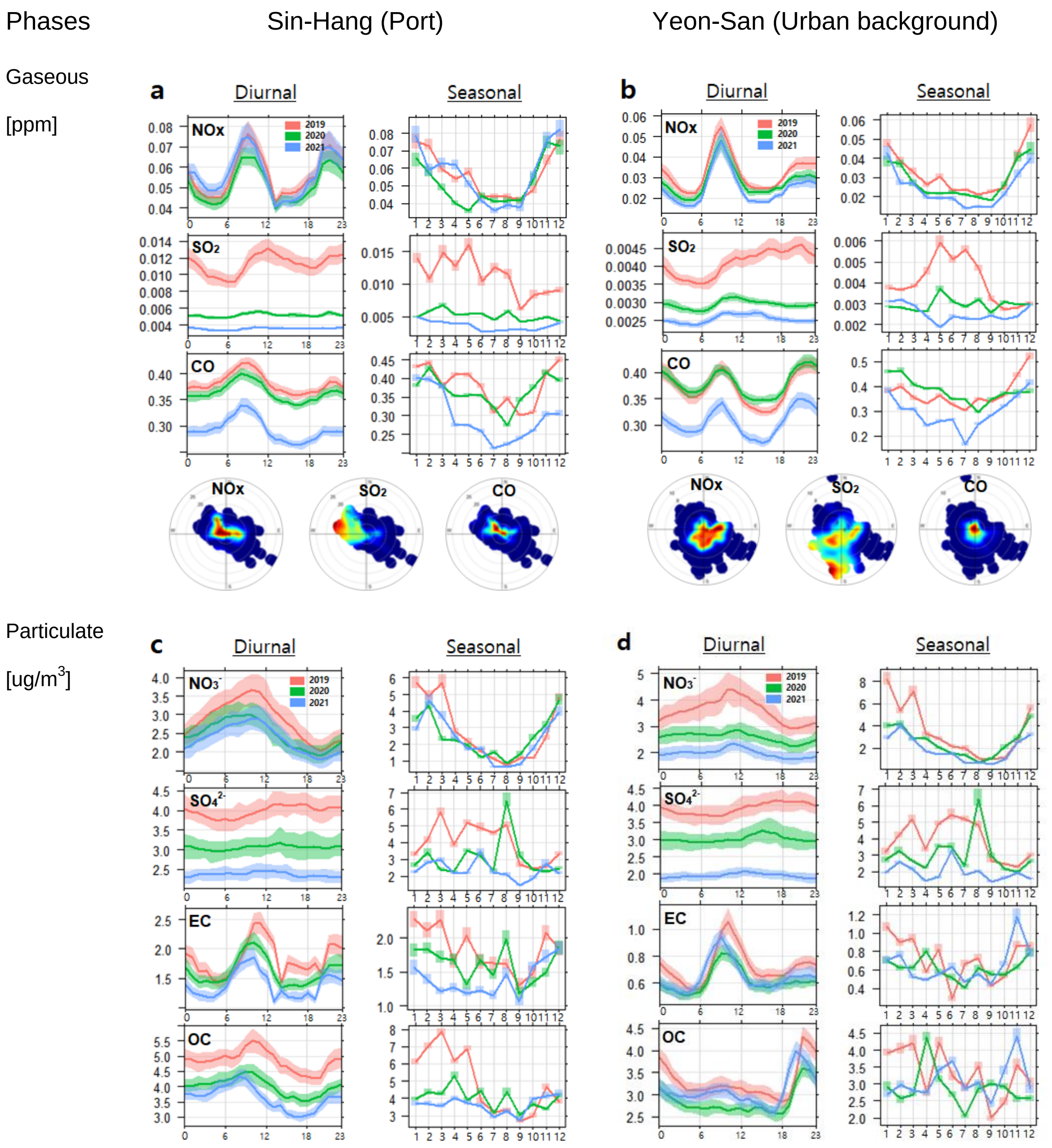


**Supplementary Figure 2. Temporal/seasonal and spatial distributions of ambient pollution level.** Concentrations of gaseous species derived from air quality monitoring stations (**a**) at port site (Sin-Hang) and (**b**) at urban background site (Yeon-San). Levels of $PM_{2.5}$ elements derived from $PM_{2.5}$ speciation network **(c)** at Sin-Hang and **(d)** at Yeon-San.

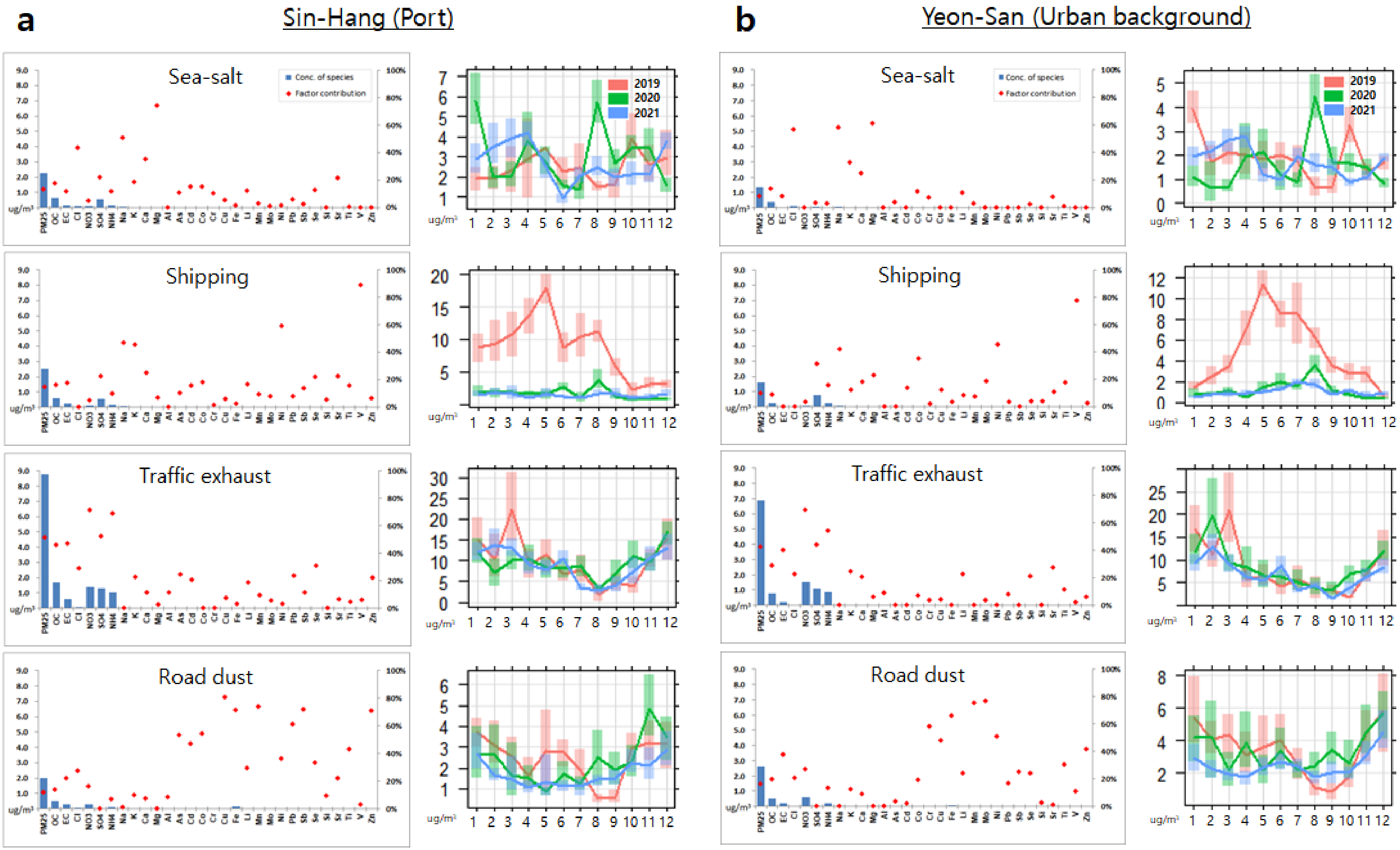
a
Sin-Hang (Port)
b
Yeon-San (Urban background)
Sea-salt
Shipping
Traffic exhaust
Road dust
Conc. of species
Factor contribution
2019
2020
2021
ug/m³
PM25
OC
EC
Cl
NO3
SO4
NH4
Na
K
Ca
Mg
Al
As
Cd
Co
Cr
Cu
Fe
Li
Mn
Mo
Ni
Pb
Sb
Se
Si
Sr
Ti
V
Zn

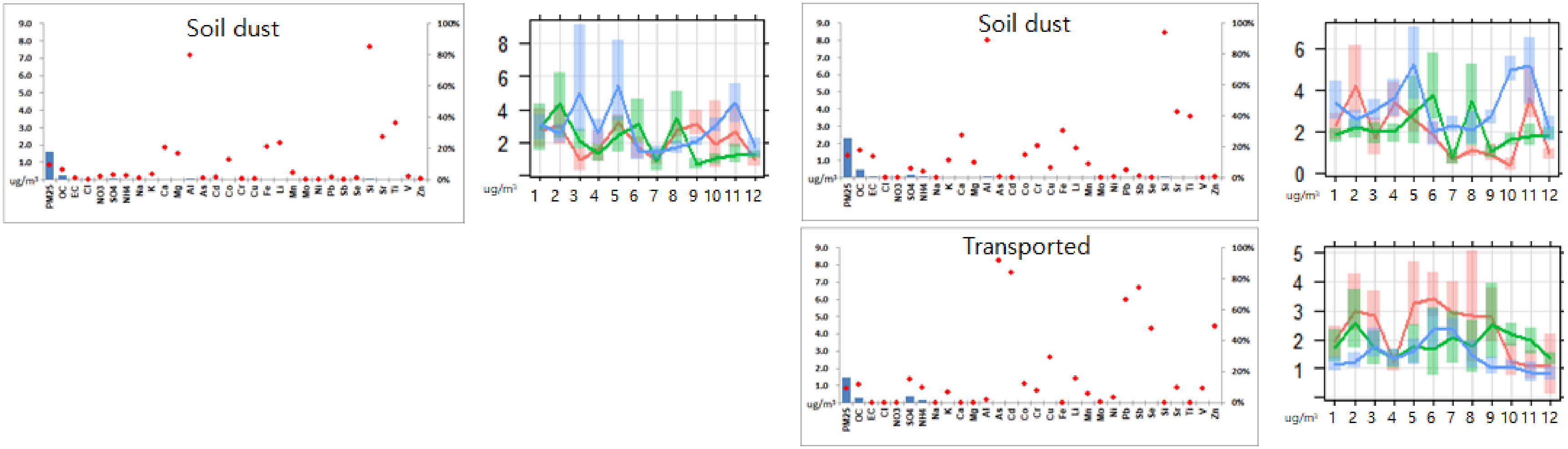


**Supplementary Figure 3. PMF factor profiles for $PM_{2.5}$ and source-specific monthly variations. (a)** $PM_{2.5}$ levels of the port dataset are determined by five major factors, and **(b)** the urban background dataset is explained by a six factor solution**.**

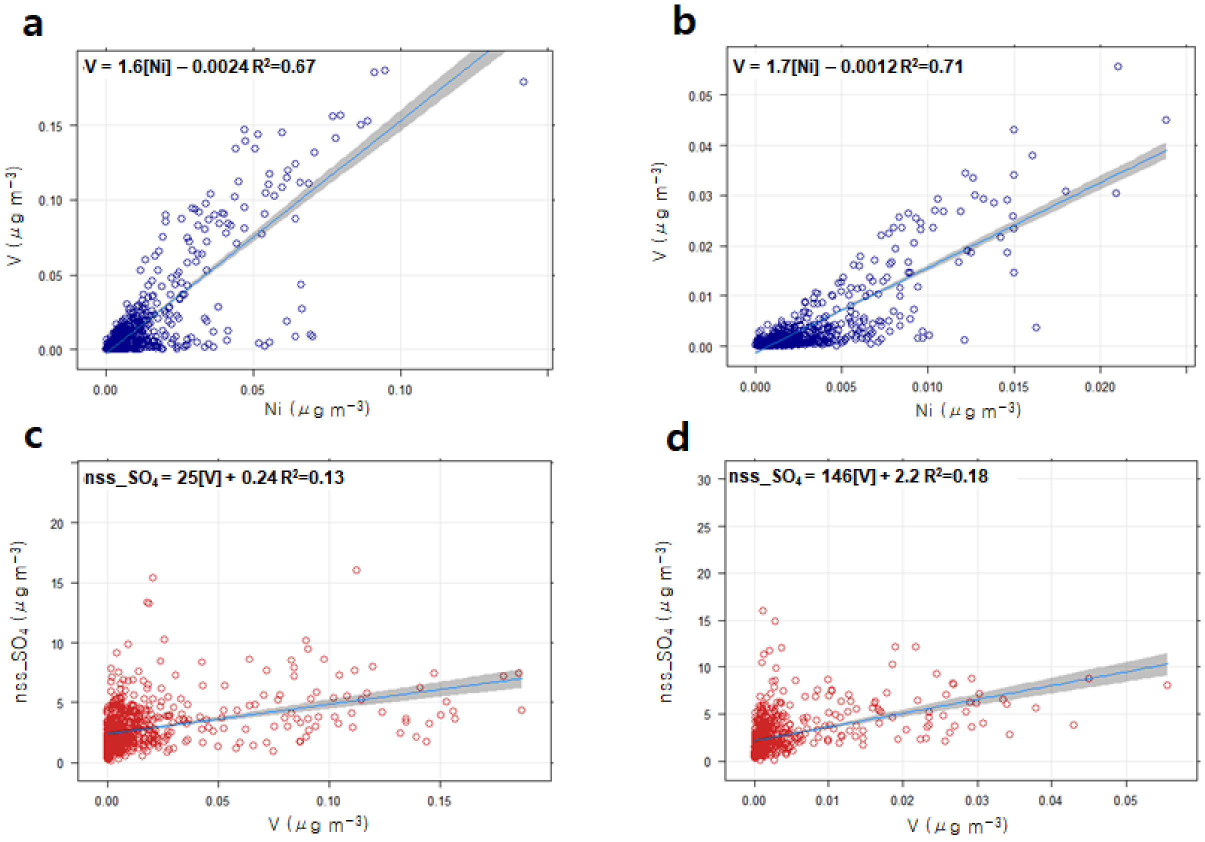


**Supplementary Figure 4. Relationships between shipping emission indicators**. **(a)** Correlation between V and Ni at Sin-Hang and **(b)** at Yeon-San. **(c)** Correlation between nss-$SO_4^{2-}$ and V at Sin-Hang and **(d)** at Yeon-San.

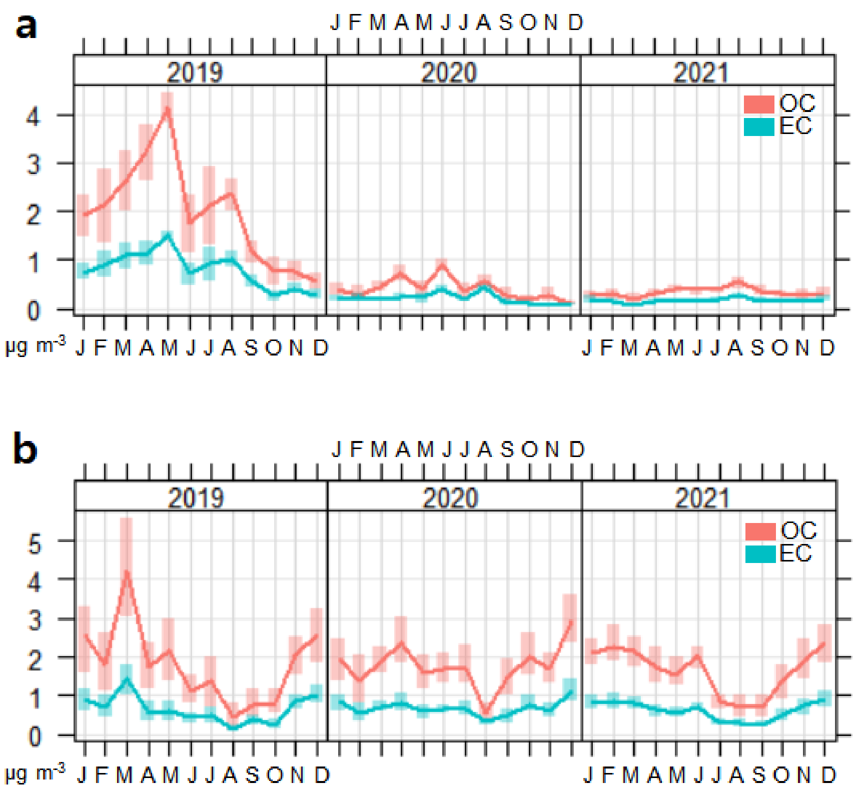


**Supplementary Figure 5. Monthly variations of PMF source-specific concentrations of carbonaceous components in $PM_{2.5}$ at a port site (Sin-Hang) between 2019 and 2021.** The concentration of organic carbon (OC) and elemental carbon (EC) associated with **(a)** shipping-source and **(b)** traffic-source.

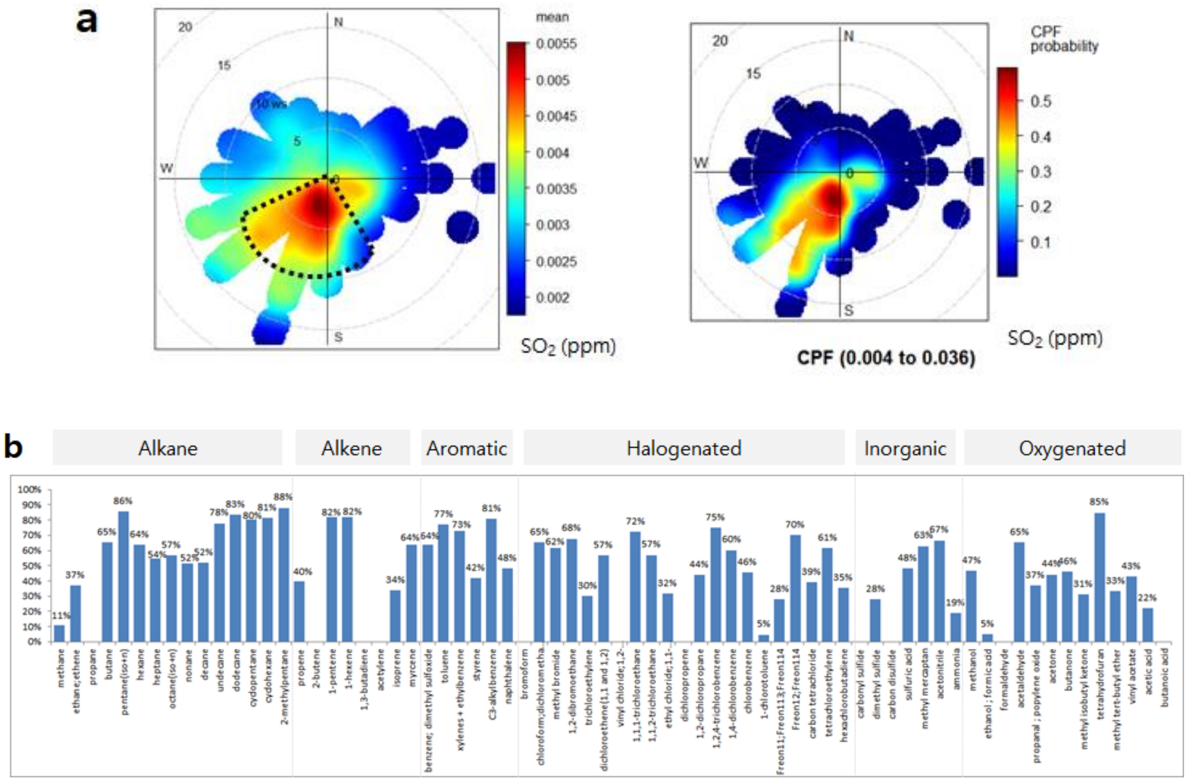


**Supplementary Figure 6. Influence of air pollutions associated with shipping emissions at *Site A*. (a)** Concentrations of $SO_2$ as a function of meteorological conditions, and a conditional probability function (CPF) plot of $SO_2$ being greater than the $70^{th}$ percentile. **(b)** Contribution profile of net shipping emissions obtained from the cross sectional spatial analysis.

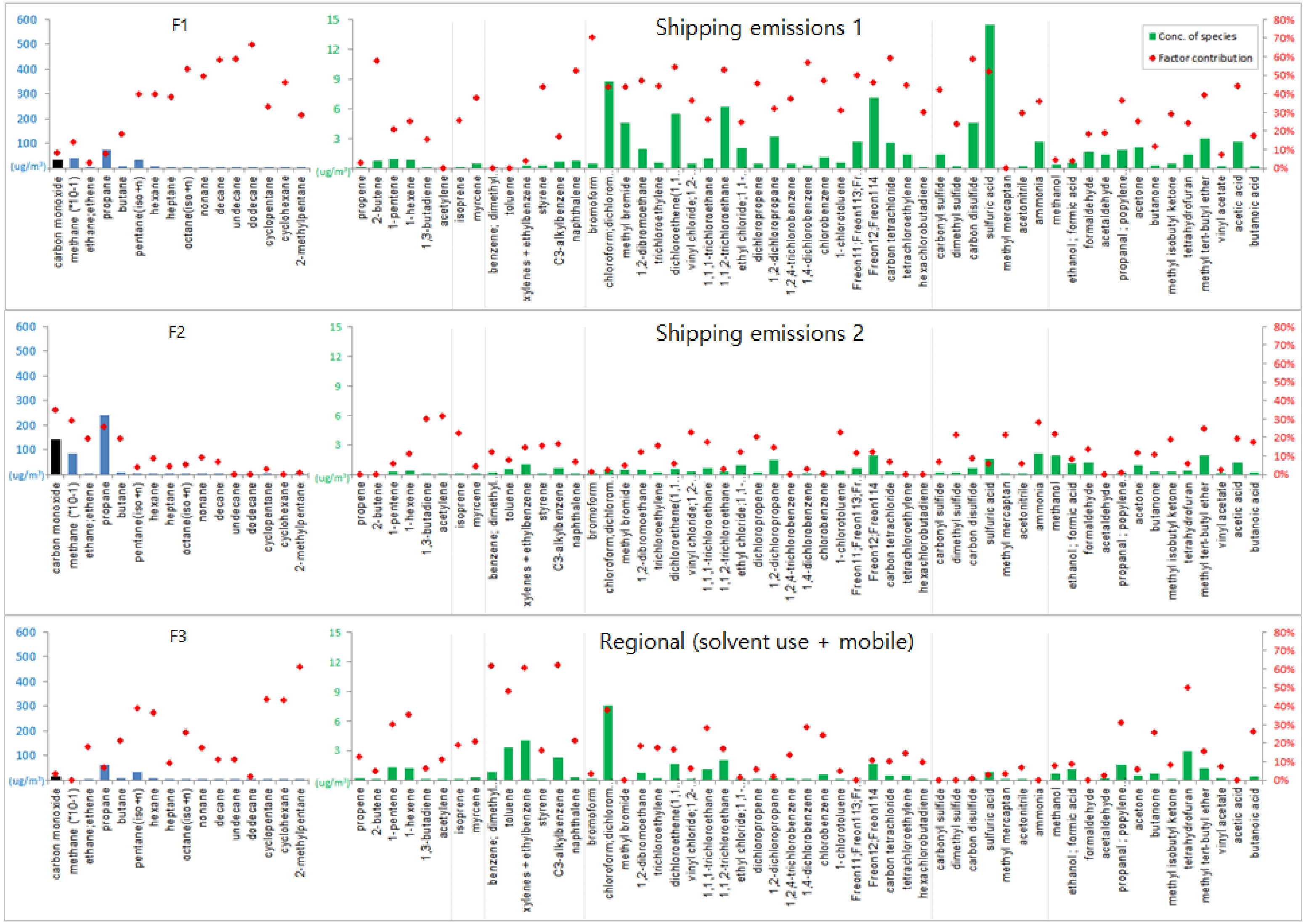
F1
Shipping emissions 1
F2
Shipping emissions 2
F3
Regional (solvent use + mobile)
Conc. of species
Factor contribution
(ug/m³)
carbon monoxide
methane (*10-1)
ethane;ethene
propane
butane
pentane(iso+n)
hexane
heptane
octane(iso+n)
nonane
decane
undecane
dodecane
cyclopentane
cyclohexane
2-methylpentane
propene
2-butene
1-pentene
1-hexene
1,3-butadiene
acetylene
isoprene
myrcene
benzene; dimethyl..
toluene
xylenes + ethylbenzene
styrene
C3-alkylbenzene
naphthalene
bromoform
chloroform;dichlorom..
methyl bromide
1,2-dibromoethane
trichloroethylene
dichloroethene(1,1..
vinyl chloride;1,2-..
1,1,1-trichloroethane
1,1,2-trichloroethane
ethyl chloride;1,1-..
dichloropropene
1,2-dichloropropane
1,2,4-trichlorobenzene
1,4-dichlorobenzene
chlorobenzene
1-chlorotoluene
Freon11;Freon113;Fr..
Freon12;Freon114
carbon tetrachloride
tetrachloroethylene
hexachlorobutadiene
carbonyl sulfide
dimethyl sulfide
carbon disulfide
sulfuric acid
methyl mercaptan
acetonitrile
ammonia
methanol
ethanol ; formic acid
formaldehyde
acetaldehyde
propanal ; popylene..
acetone
butanone
methyl isobutyl ketone
tetrahydrofuran
methyl tert-butyl ether
vinyl acetate
acetic acid
butanoic acid

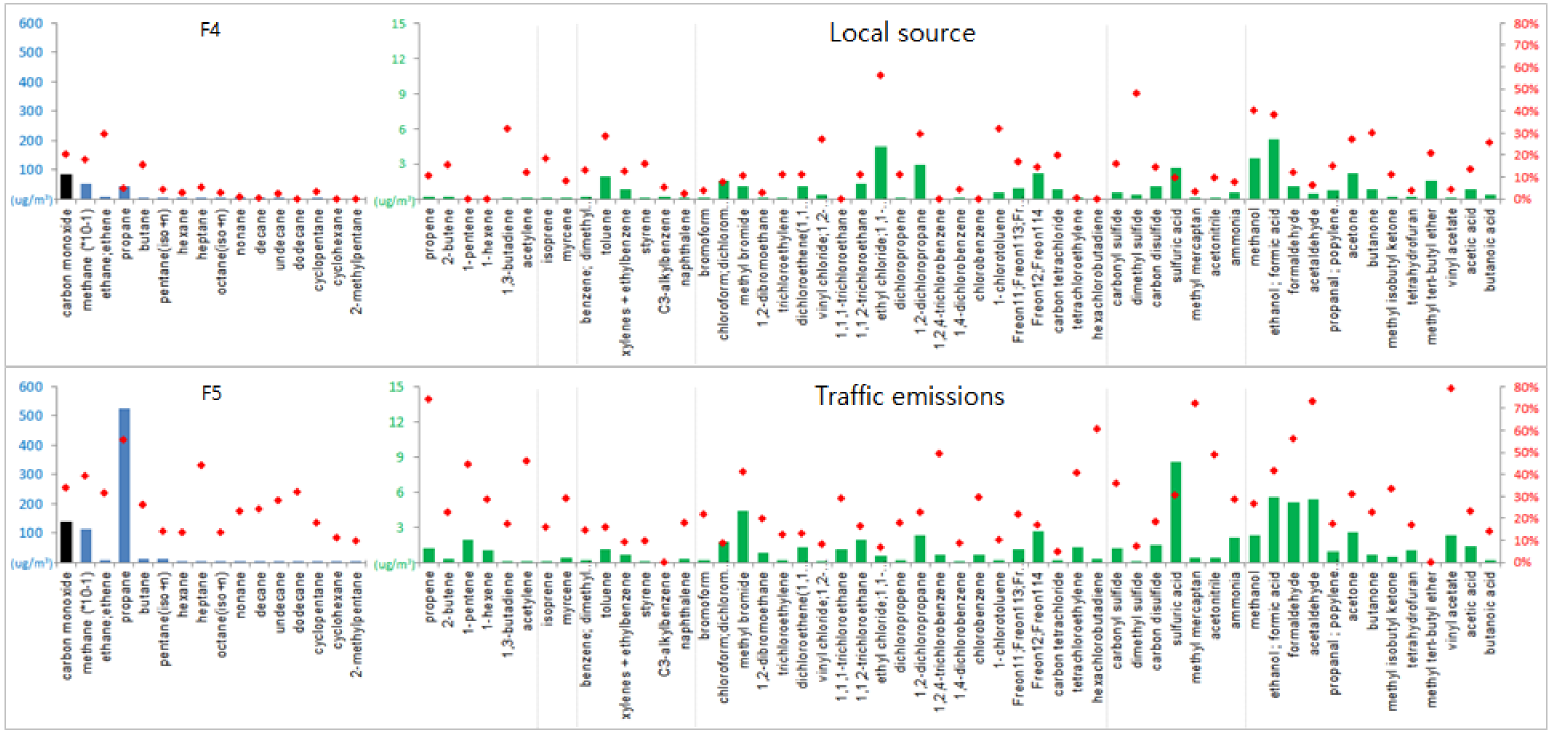


**Supplementary Figure 7. PMF factor profiles for trace gases measured by a mobile laboratory equipped with a selected ion flow tube mass spectrometery (SIFT-MS) at a port site (*Site A*).**

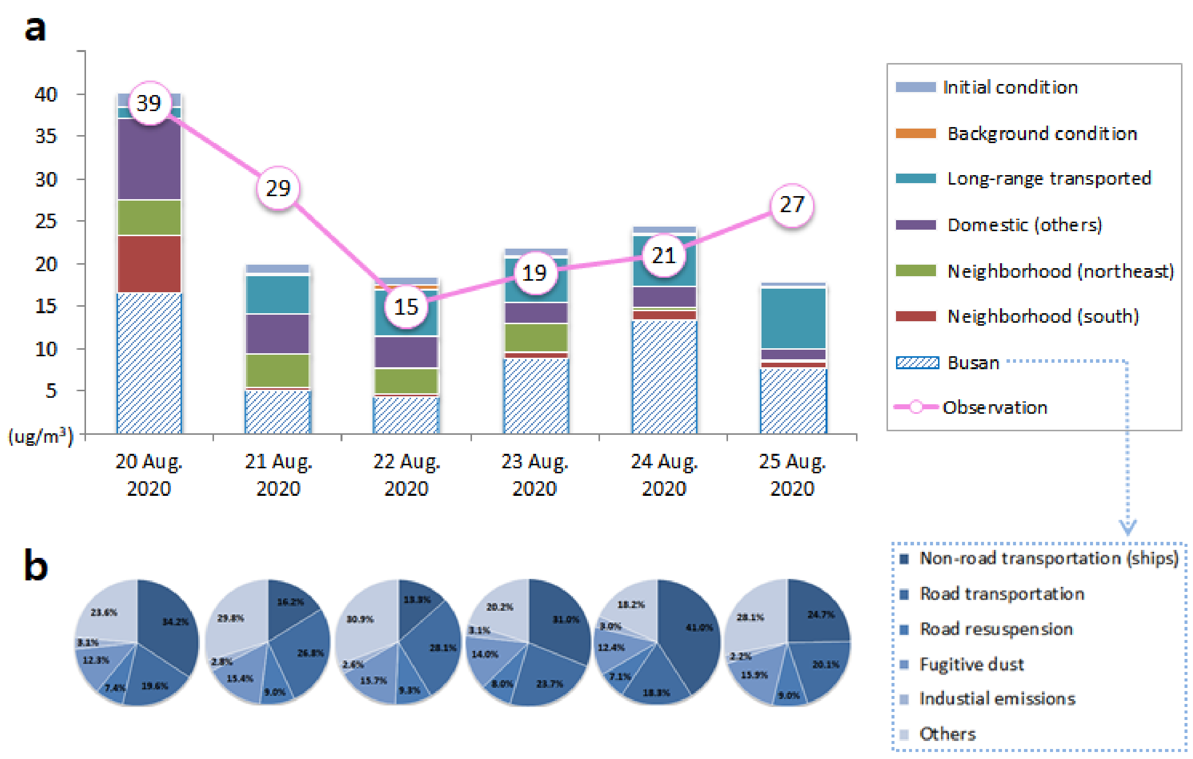


**Supplementary Figure 8. Estimated source-specific contributions to surface $PM_{2.5}$ mass concentrations at the urban background site (Yeon-San) based on CAMx/PSAT modelling results. (a)** The bar chart represents major source regions that contribute to the daily $PM_{2.5}$ levels at Yeon-San while the line chart shows the observed $PM_{2.5}$ values at the same location. **(b)** Source-specific contributions to $PM_{2.5}$ at Yeon-San attributable to local emissions in Busan.
